# Outer membrane utilisomes mediate oligosaccharide uptake in gut Bacteroidetes

**DOI:** 10.1038/s41586-023-06146-w

**Published:** 2023-06-07

**Authors:** Joshua B. R. White, Augustinas Silale, Matthew Feasey, Tiaan Heunis, Yiling Zhu, Hong Zheng, Akshada Gajbhiye, Susan Firbank, Arnaud Baslé, Matthias Trost, David N. Bolam, Bert van den Berg, Neil A. Ranson

**Affiliations:** 1 Astbury Centre for Structural Molecular Biology, Faculty of Biological Sciences, University of Leeds, Leeds, LS2 9JT, UK; 2 Biosciences Institute, The Medical School, Newcastle University, Newcastle upon Tyne NE2 4HH, UK

## Abstract

Bacteroidetes are abundant members of the human microbiota, utilising a myriad of diet- and host-derived glycans in the distal gut. Glycan uptake across the bacterial outer membrane (OM) of these bacteria is mediated by SusCD protein complexes, comprising a membrane-embedded barrel and a lipoprotein lid, that is thought to open and close to facilitate substrate binding and transport. However, surface-exposed glycan binding proteins and glycoside hydrolases also play critical roles in the capture, processing and transport of large glycan chains. The interactions between these components in the OM are poorly understood, despite being crucial for nutrient acquisition by our colonic microbiota. Here we show that for both the levan and dextran utilisation systems of *Bacteroides thetaiotaomicron*, the additional OM components assemble on the core SusCD transporter, forming stable glycan utilising machines which we term ‘utilisomes’. Single particle cryogenic electron microscopy (cryoEM) structures in the absence and presence of substrate reveal concerted conformational changes that demonstrate the mechanism of substrate capture, and rationalise the role of each component in the utilisome.

## Introduction

The human large intestine is home to a diverse microbial community, the gut microbiome, which is essential for human health^1^. Complex dietary glycans which are inaccessible to the enzymes of the human digestive tract are the primary nutrient source for the microbiome, and the utilisation of these complex sugars is essential for their survival^2,3^. This utilisation is integral to mutualism between host and bacteria, leading, for example, to the generation of short-chain fatty acids that are associated with normal gastrointestinal physiology and systemic health benefits to the host^4,5^.

The distal gut microbiome is dominated by two bacterial phyla: the Gram-positive Firmicutes, and the Gram-negative Bacteroidetes^6^. The OM of Gram-negative bacteria presents a formidable barrier to the uptake of large nutrients^7^, and gut Bacteroidetes employ a common strategy for glycan utilisation, wherein the machinery for the uptake, processing, transport and metabolism of glycans is encoded in co-regulated gene clusters known as polysaccharide utilisation loci (PULs)^8^. Glycan transport across the OM is dependent on the SusCD core components of a PUL. SusC is an integral OM, TonB-dependent active transporter, whilst SusD is a surface-exposed OM lipoprotein^8,9^. SusCD complexes exist as SusC_2_D_2_ tetramers^10–12^ (henceforth abbreviated ‘SusCD’), creating a core transportation unit with a twin barrel structure, in with each barrel is associated with its own SusD that caps the extracellular face of the transporter. Recent work suggests that SusCD transporters utilise a ‘pedal bin’ mechanism, wherein SusD undergoes hinge-like movements that alternatively expose and occlude nutrient binding sites within the SusC barrel, facilitating substrate capture and transport^12^.

Although most glycan breakdown occurs within the bacterial cell, initial binding and processing of long glycans occurs extracellularly. To achieve this, a PUL minimally encodes at least one SusCD pair, together with a surface-located, endo-acting glycanase (most commonly glycoside hydrolases; henceforth ‘GH’) and one (or more) surface glycan binding proteins (SGBPs)^8,9^. The model gut symbiont *B. thetaiotaomicron* (*B. theta*) has 88 predicted PULs, each likely dedicated to a specific glycan, but substrate specificity is known for only ~20 of them^13^. This extraordinary commitment to glycan utilisation (~20% of the *B. theta* genome) demonstrates the importance of complex glycans to bacterial survival in the distal gut.

One of the best-characterised PULs is that for levan, a plant- and bacterial-derived fructan polysaccharide of β2,6-linked fructose units with occasional β2,1 branches^14,15^. We previously characterised the binding and uptake of fructo-oligosaccharides (FOS) by the core SusCD levan transporter (SusCD^lev^)^10,12^. However, important steps preceding FOS capture and transport remain unclear. Transport-competent FOS are generated from levan by a surface-exposed lipoprotein, a GH32-family levan endo-glycanase (GH^lev^)^14,16^, while an SGBP (SGBP^lev^) is thought to recruit levan at the cell surface^14,17^. A key unresolved question is whether and how these additional lipoproteins associate with their SusCD transporters to facilitate glycan processing and transport. In the archetypal starch PUL, which encodes a SusCD transporter, two SGBPs (SusE and SusF) and a surface amylase (SusG)^18,19^, seminal studies suggested that SusCD and SGBP form a complex^19^. However, recent studies indicate that more dynamic and transient, substrate-induced complexes are formed ^20^, with the SGBPs acting as immobile starch binding centres around which SusCD transporters and the amylase assemble^21^. In contrast, the SusCD pair from an uncharacterised PUL in *B. theta* forms a stable complex with two lipoproteins of unknown function^10^. Thus, two broad models exist: (1) components transiently assemble in response to substrate, and (2) all components form a stable complex in the OM regardless of the presence of substrate.

**Extended Data Figure 1:**
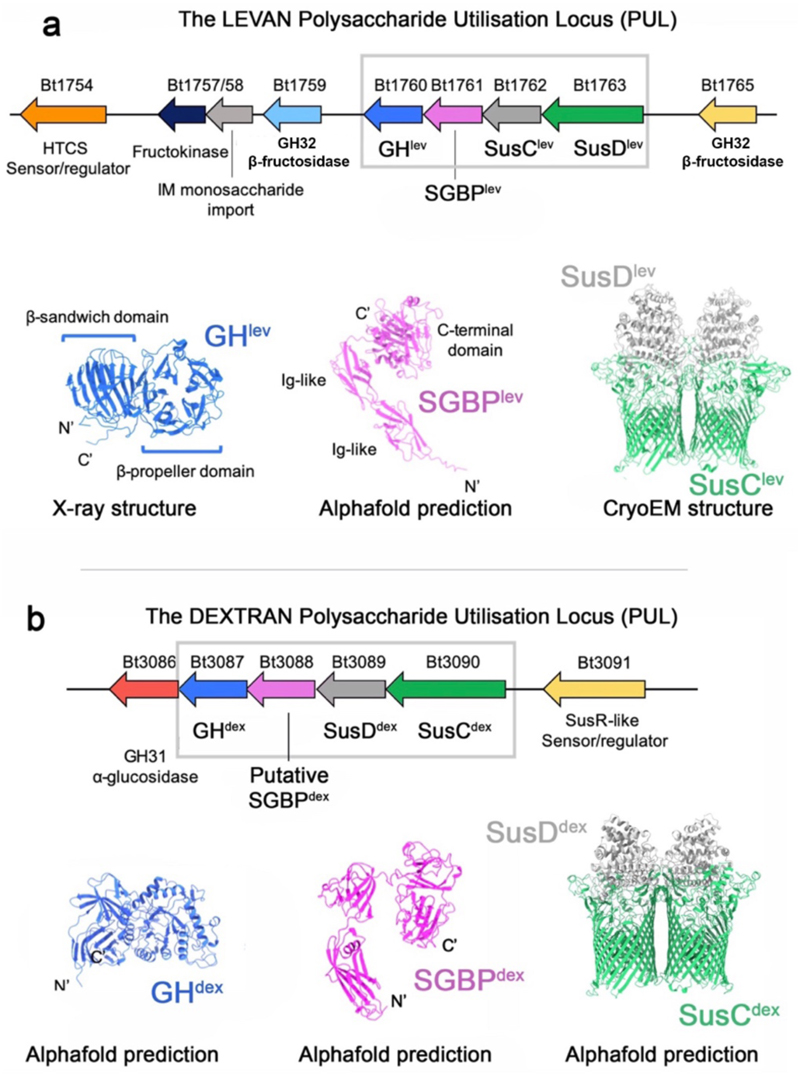
The levan and dextran polysaccharide utilisation loci. **(a)** Organisation of the levan PUL showing relative gene positions within the PUL, with functions indicated. The four OM-associated PUL components (SusC^lev^, SusD^lev^, GH^lev^ and SGBP^lev^) are highlighted by the grey box. An X-ray structure of GH^lev^ (Bt1760; GH32 endo-levanase) is shown (blue; PDB-ID: 6R3R). The AlphaFold2-predicted model for SGBP^lev^ (Bt1761) is shown (pink) oriented such that the N-terminus is at the bottom and the proposed (C-terminal) levan binding domain is at the top. Note that the N-termini of GH^lev^ and SGBP^lev^ will be lipidated and associated with the outer leaflet of the OM. The cryo-EM structure of the dimeric SusCD^lev^ complex in its open-open state is shown (SusC^lev^ is green, SusD^lev^ is grey). **(b)** Organisation of the dextran PUL showing gene positions within the locus with functions labelled. OM-associated PUL components are boxed in grey. AlphaFold2-predicted models for GH^dex^ (Bt3087; GH66 endo-dextranase), the putative SGBP^dex^ (Bt3088) and the SusCD^dex^ complex, are shown coloured as for the levan PUL in (**a**).

Here we show, using quantitative proteomics and single-particle cryo-EM, that all four OM components of the levan PUL from *B. theta* (ED Fig. 1a) exist in a stable complex that we term a ‘utilisome’ in keeping with the “PUL” acronym. The four OM components of the *B. theta* dextran (an α1,6-linked glucose polymer with occasional α1,3 glucose branches) PUL (ED Fig. 1b) adopt a similar architecture, suggesting that utilisomes may be a generic feature of PULs in the Bacteroidetes. Upon addition of substrate, the levan utilisome undergoes large conformational changes, revealing the substrate-bound states of GH^lev^ and SGBP^lev^, and demonstrating that the pedal-bin mechanism of nutrient capture operates in the presence of all OM-localised components of the PUL. Collectively, these data show that utilisomes constitute multi-component, macromolecular machines on the cell surface, the architecture of which is consistent with efficient capture, processing, and transport of complex glycans.

## Results

### Additional lipoproteins associate with the core transporter in the absence of substrate

**Extended Data Figure 2:**
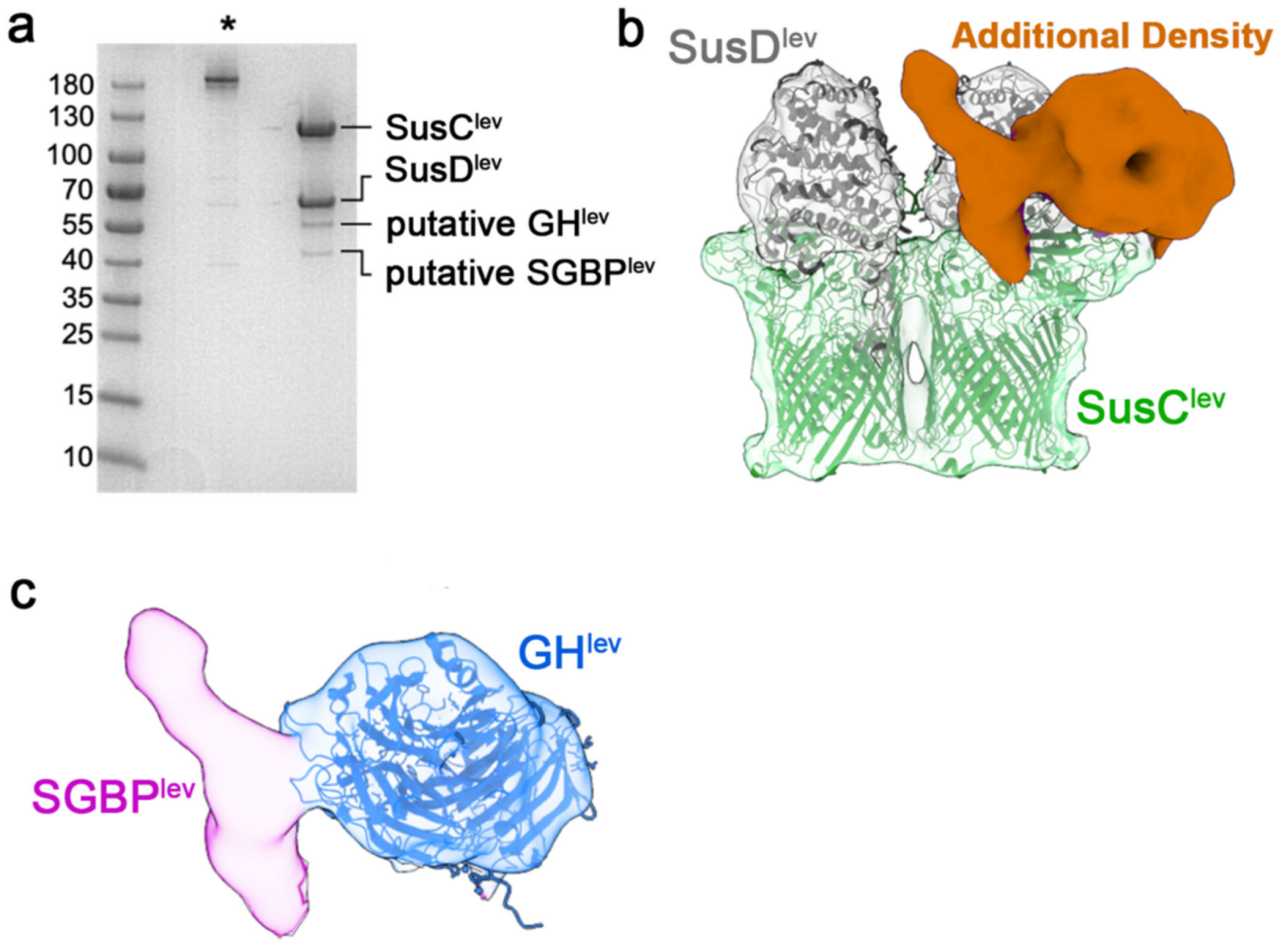
Additional lipoproteins co-purify with the SusCD core. **(a)** SDS-PAGE of the previously-studied sample of LDAO-purified SusCD^lev 10^ before (asterisk) and after boiling. The boiled sample shows two weak bands in addition to those for SusC^lev^ and SusD^lev^, which were subsequently identified as GH^lev^ and SGBP^lev^ by mass spectrometry. (**b**) A class average obtained during 3D classification of the levan SusC_2_D_2_ core complex. The SusC and SusD components (green and grey respectively) are docked into the density. A large region of density remains unassigned (orange). (**c**) Isolated view of the previously unassigned density with the crystal structure of GH^lev^ (blue cartoon) fitted into the EM density (blue) as a rigid body. The remaining density was therefore attributed to SGBP^lev^ and is coloured magenta.

**Figure 1:**
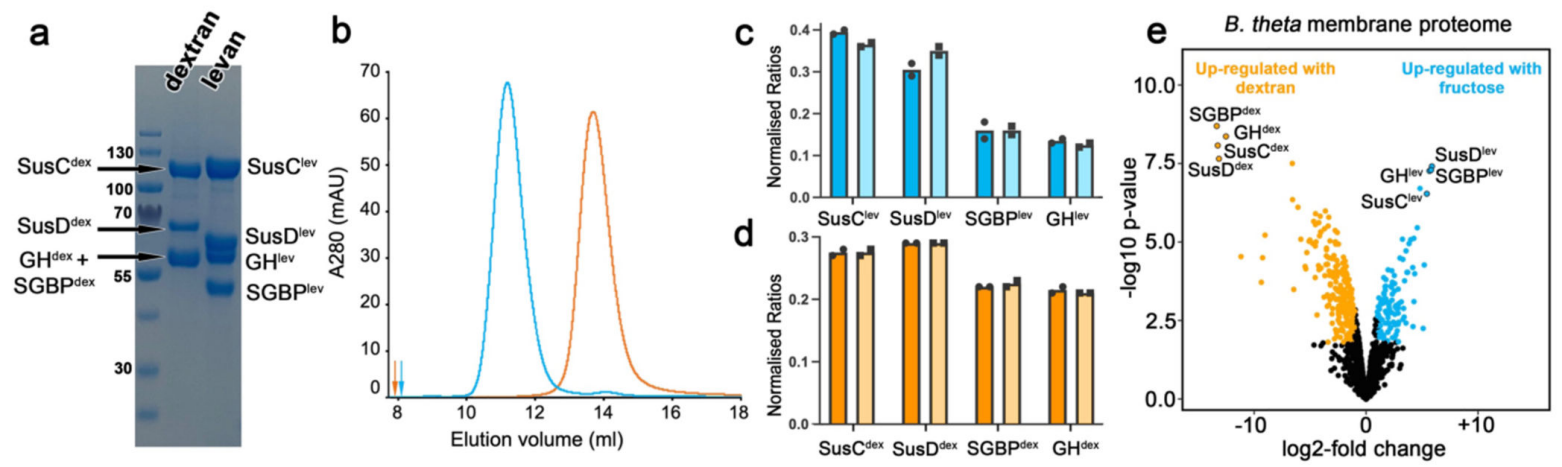
Stable 4-component complexes in the OM of *B. thetaiotaomicron*. **(a)** SDS-PAGE of the purified levan and dextran utilisome complexes. (**b**) Analytical SEC elution profiles for the levan (blue) and dextran utilisomes (orange), analysed on Superdex-200 and Superose-6, respectively (void volumes indicated by arrows). OM abundance of the four components of (**c**) the levan (blue) and (**d**) the dextran (orange) systems obtained from fructose- and dextran-grown cells, respectively (dark coloured bars). The light-coloured bars show the normalised abundance of detergent-purified levan or dextran 4CCs spiked into the proteome samples of dextran- or fructose-grown cells. The dots show the individual values of two independent replicates. (**e**) Volcano plot of the *B. thetaiotaomicron* OM proteome from fructose versus dextran grown cells.

Previously, we used single particle cryoEM to capture the SusCD levan transporter (SusCD^lev^) in several conformational states^12^. During structure determination, a minor subset (~10%) of the dataset was identified that contained additional density (ED Fig. 2). SDS-PAGE (and mass spectrometry) revealed co-purification of sub-stoichiometric amounts of GH^lev^ and SGBP^lev^ (ED Fig. 2a). Given this, we docked the crystal structure of *E. coli*-expressed, inactive GH^lev^ (PDB ID 7ZNR; Supplementary Table 1 and Methods) into the map, which fitted well into part of the unassigned density (ED Fig. 2b,c), leading us to assign the remainder to the SGBP^lev^, for which no structure was available. The additional density was associated with only one of the two available SusC^lev^ barrels, and this seemed unlikely to represent an intact complex given the dimeric nature of SusCD^lev^. After moving the His_6_-tag from SusD^lev^ to the C-terminus of SGBP^lev^, and using milder extraction detergent (methods), we were able to purify a complex containing approximately stoichiometric amounts of all four components, for both the levan complex and a related system for dextran utilisation (Fig. 1a,b).

### OM-localised PUL components exist as stoichiometric four-component complexes

Successful purification of two different detergent-solubilised four-component complexes strongly suggests that the complexes are stable in the OM. To shed light on the relative abundance of the components, we performed quantitative proteomics using intensity-based absolute quantification (iBAQ)^22^ on outer membrane fractions from *B. theta* grown on fructose or dextran. While the additional lipoproteins of the putative levan four-component complex (4CC) appear to be substoichiometric (dark bars, Fig. 1c), SDS-PAGE and the monodisperse peaks in size exclusion chromatography (Fig. 1a,b) suggest that the iBAQ analysis underestimates the abundance of these additional lipoproteins in the OM. For the dextran complex (obtained from *B. theta* grown on dextran), the four components are present in roughly equimolar amounts based on the iBAQ analyses (dark bars, Fig. 1d).

As expected from transcription studies^14^, the four OM components of the levan PUL are among the most-highly expressed membrane proteins in *B. theta* when grown on minimal media with fructose as the sole carbon source, and dextran PUL components were not detectable (Fig. 1e). Conversely, in minimal media with dextran, the dextran PUL components are highly expressed and levan PUL components are present at just ~1 % of the level found when cells are grown on fructose. This allowed us to spike membrane proteome samples from cells grown on fructose or dextran with detergent-purified dextran or levan 4CCs, respectively. For both systems, the relative abundance of the four components in the spiked versus endogenous membrane samples was very similar (light versus dark bars; Fig. 1c,d), suggesting no excess of any component exists in the OM. Collectively these data support the hypothesis that OM-localised PUL components are present as equimolar 4CCs, which we term ‘utilisomes’ in keeping with the 'PUL' acronym.

### Single-particle cryo-EM reveals an octameric, four-component levan utilisome

**Extended Data Figure 3:**
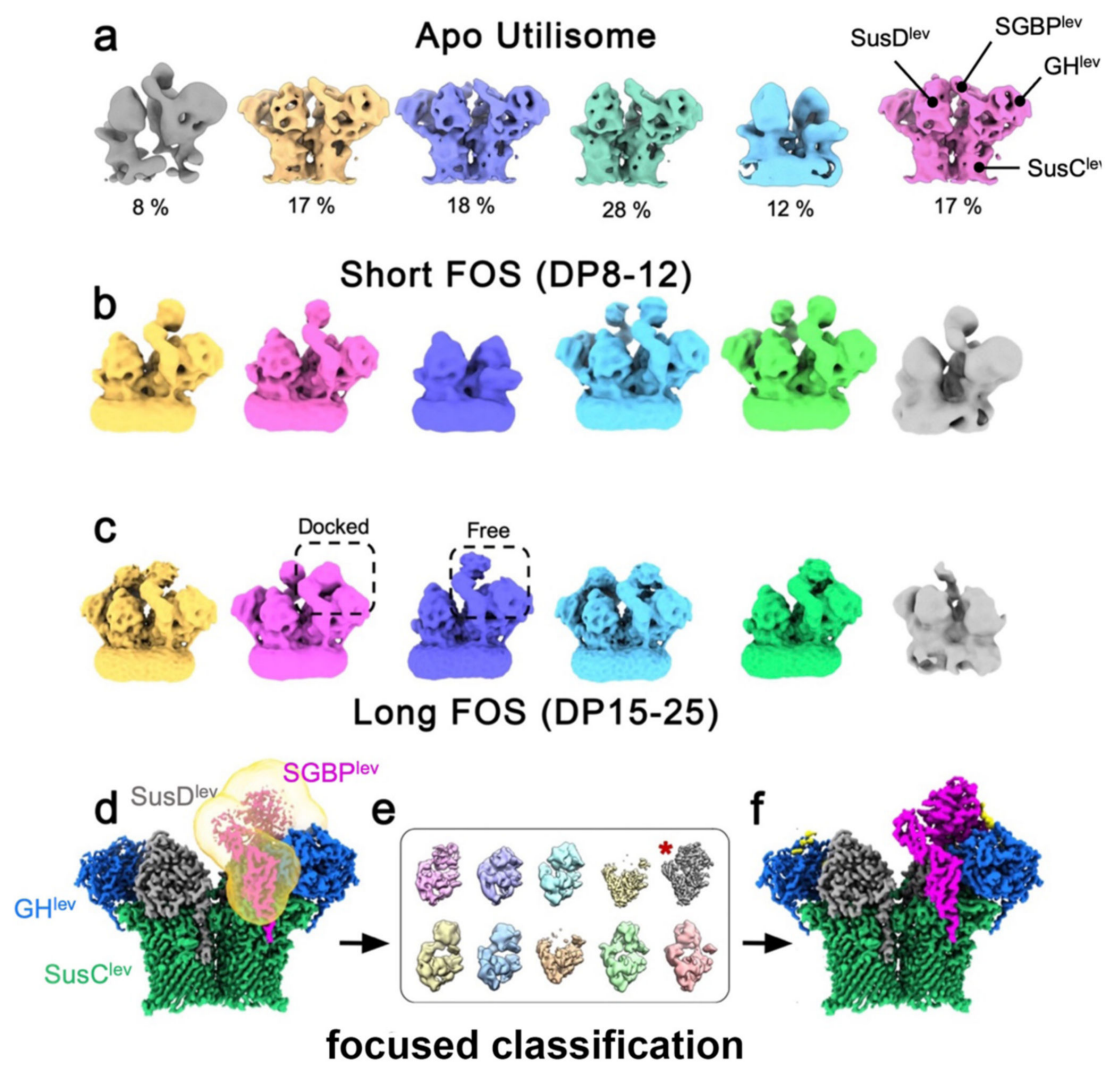
Classification of levan utilisome Data. (**a**) Output of the first round of 3D classification for apo utilisome data. Yellow, purple and pink classes represent the octameric complex *i.e*. the complete octameric utilisome. The green class shows the additional ipoproteins associated with just one SusC unit whilst the blue class shows that a small proportion of SusC_2_D_2_ core complex was present. (**b**) Output of 3D classification for the levan utilisome with an active levanase in the presence of FOS DP8-12. Classes (viewed in the plane of the membrane) containing particles of the complete octameric complex were observed (blue and green) as well as hexameric complexes containing a single copy of the SGBP^lev^ and GH^lev^ (pink and yellow). A class containing SusCD^lev^ in isolation is also present (purple). (**c**) Outputs of 3D classification for long FOS (DP15-25) showing that SGBP^lev^ can adopt a ‘docked’ conformation proximal to both the SusD and levanase. (**d**) A consensus refinement of all classes containing at east one docked SGBP (yellow, pink, cyan and green in panel (**c**)). A mask was created around the region of interest (transparent yellow). (**e**) Outputs of focused classification on the masked region without alignment. A class displaying high resolution for the region of interest is marked with a red asterisk. Independent half maps were reconstructed using unmasked particles belonging to this class. (**f**) Sharpened reconstruction generated with the aforementioned half maps showing improved density for SGBP^lev^.

**Figure 2:**
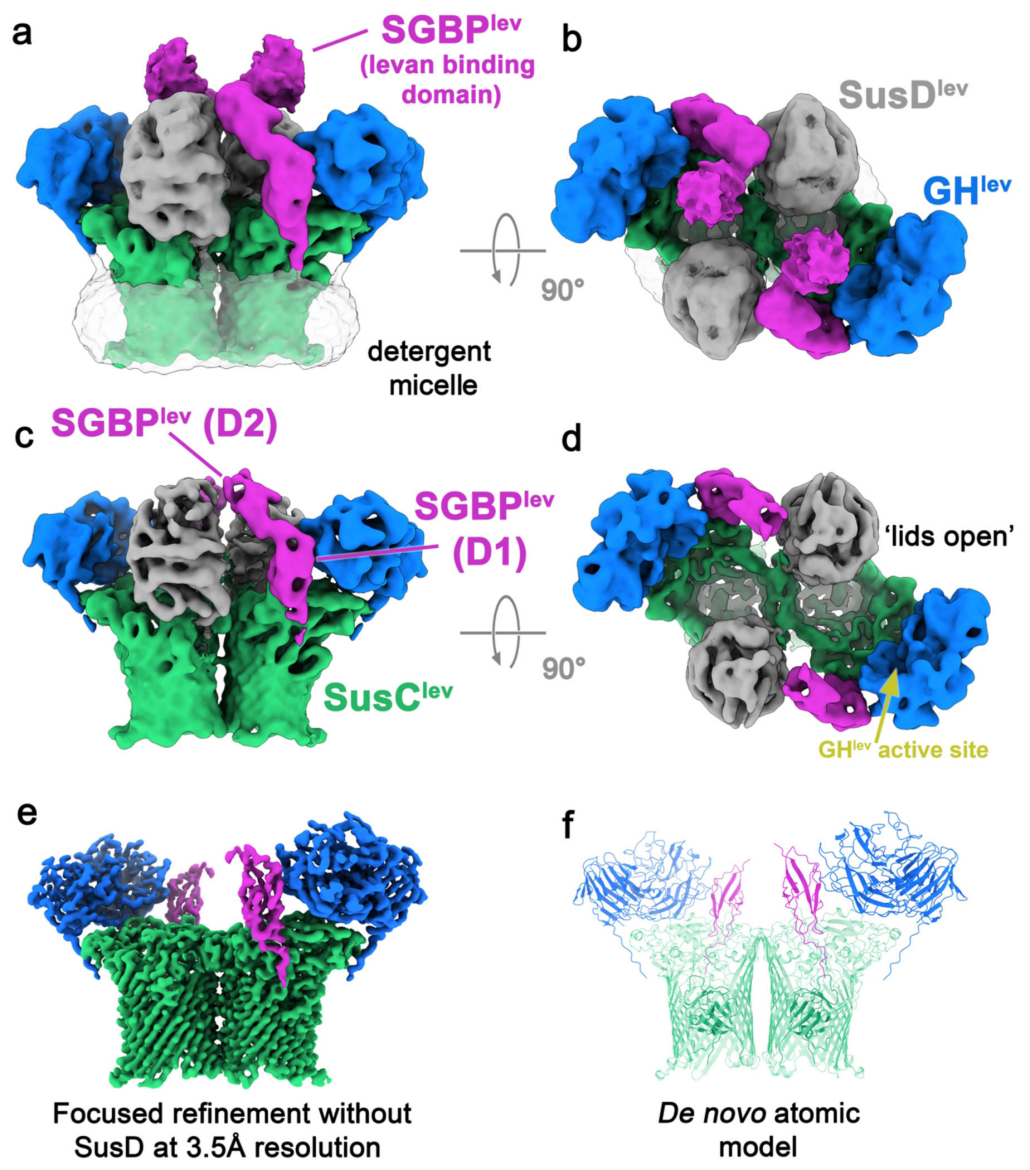
The organisation of the apo levan utilisome revealed by cryo-EM. (**a**) A side view (in the plane of the OM), and (**b**) top view (perpendicular to the OM plane from outside the cell) of a ~6 Å 3D refinement of one conformational state of the apo utilisome obtained by 3D classification, shown at low threshold. SusC^lev^ is green, SusD^lev^ is grey, GH^lev^ is blue, SGBP^lev^ is magenta, and the weak density for the detergent micelle is transparent grey. (**c**) and (**d**) show identically positioned and coloured views at high threshold. The position of the GH^lev^ active site is shown by a yellow arrow in (**d**). (**e**) C2 symmetrized reconstruction at 3.5 Å from a focused refinement, with data masked to exclude the variable SusD subunit positions. Note that all particles in this dataset possess SusD components, and their absence here is a result of focused refinement. (**f**) *De novo* atomic model built into the density shown in (**e)**.

The structure of the DDM-solubilised, substrate-free levan utilisome was assessed by single particle cryo-EM, and 2D class averages showed clear evidence for additional components (ED Fig. 3a). Subsequent 3D classification revealed ~52 % of the particles contain a SusCD^lev^ core transporter, as well as two copies of both GH^lev^ and SGBP^lev^ (Figure 2a-d). We propose that this octameric complex with two copies of each of the four components represents the complete levan utilisome. The remainder of the dataset corresponded to utilisome sub-complexes comprising a SusCD^lev^ pair, with a single copy of GH^lev^/SGBP^lev^ (~28 %), a small population of ‘naked’ SusCD^lev^ dimers (~12 %), and some ‘junk’ particles where SusC barrels are poorly defined (~8%) (ED Fig. 3a). Based on the proteomics and biochemical data, we believe that the substoichiometric classes are the result of partial denaturation of the complex occurring in the thin-film environment that is present during grid preparation.

### The architecture of the levan utilisome suggests concerted function

**Extended Data Figure 4:**
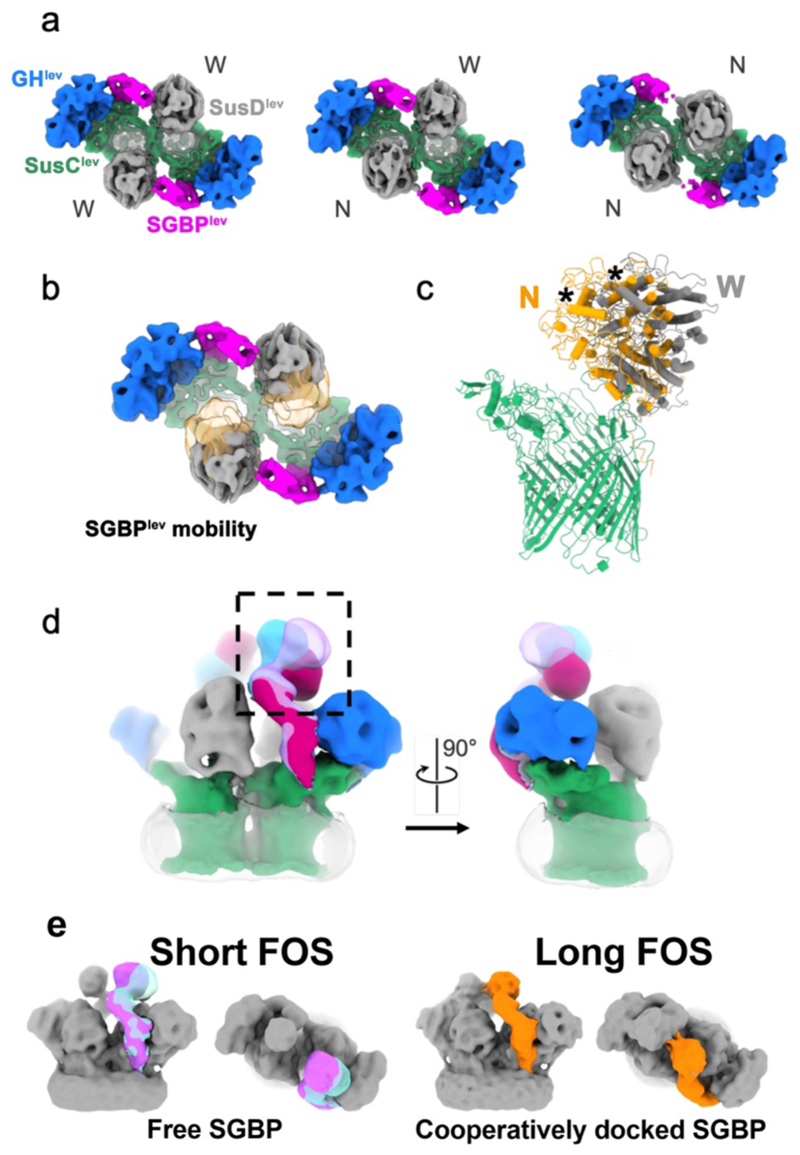
Conformational variability in apo and substrate-bound levan utilisomes. (**a**) 3D classification of apo levan utilisome viewed from outside the cell. SusC^lev^ (green), SusD^lev^ (grey), SGBP^lev^ (magenta), and GH^lev^ (blue). Classes are separated on their SusD^lev^ lid positions. Wide-wide (WW), normal-wide (NW), and normal-normal (NN) open states (from left to right) (**b**) Overlay of the wide (SusD grey) and normal (SusD orange) open states of the complex. (**c**) Overlay of atomic models for the normal versus wide open state generated by a rigid-body fit of SusD^lev^ into the cryoEM density. A monomer is shown for clarity and an asterisk marks the same SusD^lev^ helix in both models. (**d**) A view of the utilisomes shown at high threshold in the plane of the membrane (left). Different conformations of the SGBP^lev^ observed in 3D classification are overlaid to demonstrate the flexibility of this subunit (boxed region). The same view rotated 90° is shown (right). Disordered micelle density is shown as translucent grey. **(e)** Variability of the SGBP^lev^ position in the substrate-bound utilisomes with short FOS (~DP8-12) and an active GH^lev^, and long FOS (DP15-25) with an inactive GH^lev^. A novel state is uniquely observed in the long FOS structure with one SGBP^lev^ (orange) reaching across and contacting the SGBP^lev^ associated with the other SusC subunit that is present in a docked state. This conformation is consistent with both SGBP^lev^ subunits in the utilisome interacting with the same chain of substrate.

The structure of the apo levan utilisome was initially assessed using 3D classification. We identified two regions of conformational heterogeneity: the C-terminal domain of SGBP^lev^, and in the position of the SusD^lev^ lids (ED Fig. 4a-d; Methods). All complexes within this dataset have open lids, but they are open to differing degrees. Within a complex, the lids appear to operate independently, such that differing lid positions break the two-fold symmetry of the complex (ED Fig. 4a-c). Refinement of particle stacks corresponding to differing SusD^lev^ lid positions gave reconstructions that were limited to ~6 Å. In contrast to our findings for the LDAO-solubilised SusCD^lev^ core complex^10,12^, all SusC^lev^ barrels contain density for the plug domain that occludes the barrel. Thus, SusCD^lev^ transporters are open, plugged, and competent to accept substrate. Despite the conformational heterogeneity in the position of the SusD^lev^ lid, SusC^lev^, GH^lev^, and the N-terminal domain of SGBP^lev^ constitute a rigid unit with two-fold (C2) symmetry. Application of a mask that excluded the density for the SusD^lev^ lids followed by focused refinement allowed inclusion of all particles, regardless of SusD^lev^ position, and resulted in a map with a global resolution of 3.5 Å (Fig. 2e), allowing us to refine models for SusC^lev^ and GH^lev^, and to build *de novo* the N-terminal domain of SGBP^lev^ (Fig. 2f).

**Extended Data Figure 5:**
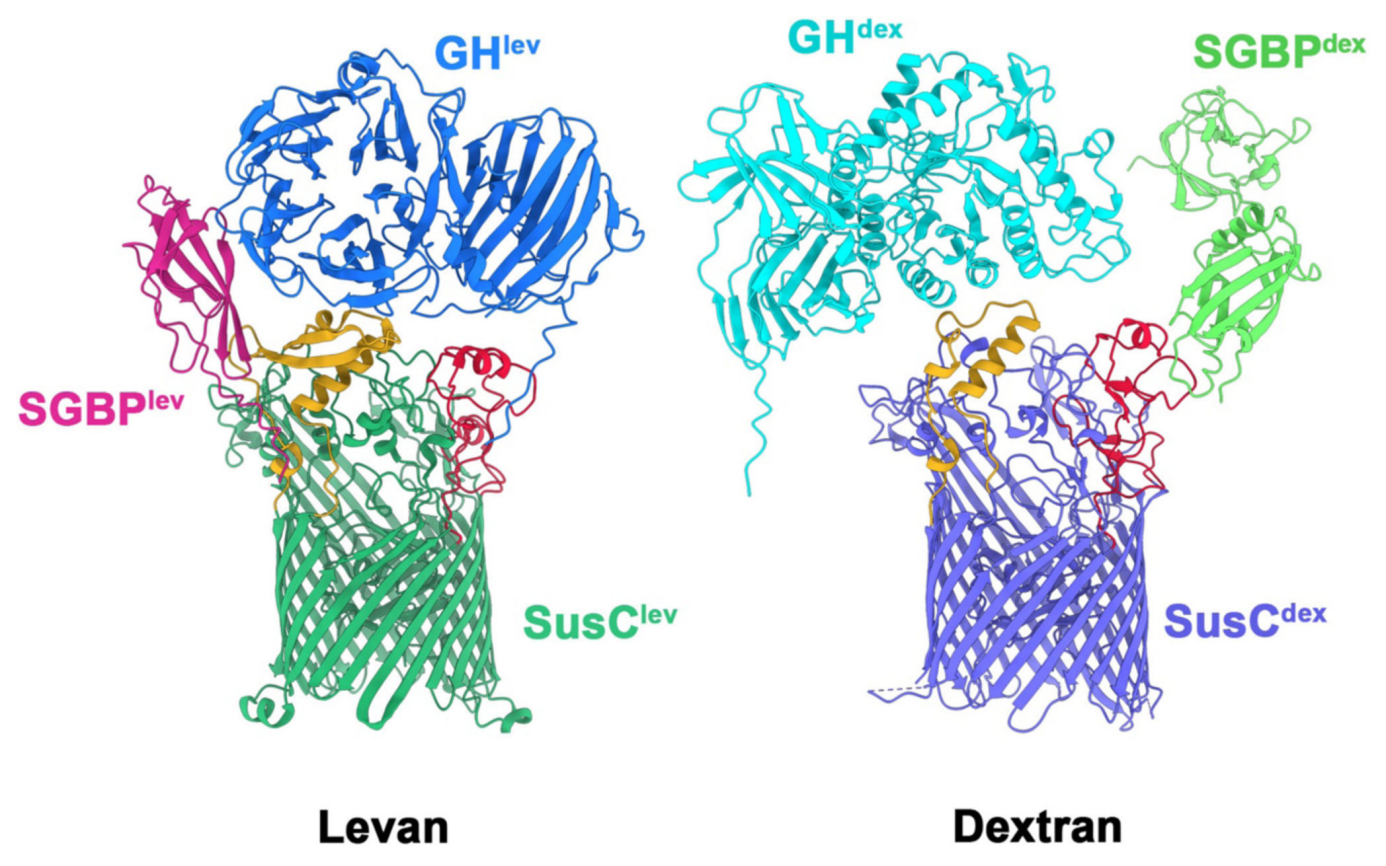
SusC extracellular loops that contribute to lipoprotein interface interactions differ in the levan and dextran utilisomes. **a**, Arrangement of GH^lev^ and SGBP^lev^ on SusC in the levan utilisome. GH^lev^ makes contacts with extracellular loop 1 (gold) and extracellular loop 9 (red), while SGBP^lev^ only makes contacts with extracellular loop 1. **b**, Arrangement of GH^dex^ and SGBP^dex^ on SusC in the dextran utilisome. Here, extracellular loop 1 of SusC^dex^ is the primary site of interaction for GH^dex^, while extracellular loop 9 comprises the interface with SGBP^dex^. For clarity, one half of the utilisome is shown in each case, and SusD components are omitted. Note that the dextran utilisome model is a composite of cryo-EM structures (SusC^dex^) and predicted models from AlphaFold2 (GH^dex^ and SGBP^dex^).

The refined model for GH^lev^ within the utilisome is similar to the crystal structure of *E. coli*-expressed GH^lev^ solved in isolation (PDB: 7ZNR)(Cα rmsd = 0.54 Å). GH^lev^ is mounted on the lip of the SusC^lev^ barrel, with extracellular loops 1 and 9 of SusC^lev^ contributing to the binding interface (ED Fig. 5). This GH^lev^ binding occurs at the opposite side of SusC^lev^ from the SusD^lev^ binding site, in a position that does not obviously impede closure of the SusD^lev^ lid (Figure 2a-d). The GH^lev^ active site is positioned close to the mouth of the SusCD^lev^ transporter (minimum distance ~30 Å; see methods), consistent with concerted function with the transporter (Figure 2d). SGBP^lev^ is adjacent to GH^lev^ on the lip of SusC^lev^ (see Figure 2b,d); ED Fig. 5). An AlphaFold2 model^23^ of SGBP^lev^ predicts two N-terminal Ig-like domains, and a C-terminal levan binding domain (ED Fig. 1a)^24^. In this substrate-free utilisome structure, there is clear density for only the two N-terminal, Ig-like domains with the second being weaker than the first. Poorly-resolved density is present for the C-terminal, levan-binding domain which extends away from the transporter, towards the extracellular space, with diffuse density visible only at high threshold levels (Fig. 2a,b). The lip of SusC therefore serves as a platform for the additional lipoproteins, perhaps explaining why SusC transporters are ~40 % larger than classic, monomeric TonB dependent transporters^9^. Collectively, these data provide important insight into how the utilisome architecture may contribute to efficient levan utilisation. The C-terminal domain of SGBP^lev^ projects away from the cell, where it would facilitate capture of levan chains from the environment. The flexibility of the SGBP^lev^ (ED Fig. 4d) may facilitate processing of those levan chains at the adjacent GH^lev^, and any cleavage products would be released close to their binding site at the SusCD^lev^ interface, promoting efficient loading of the transporter.

### A stable, four-component utilisome is not unique to the levan system

**Extended Data Figure 6:**
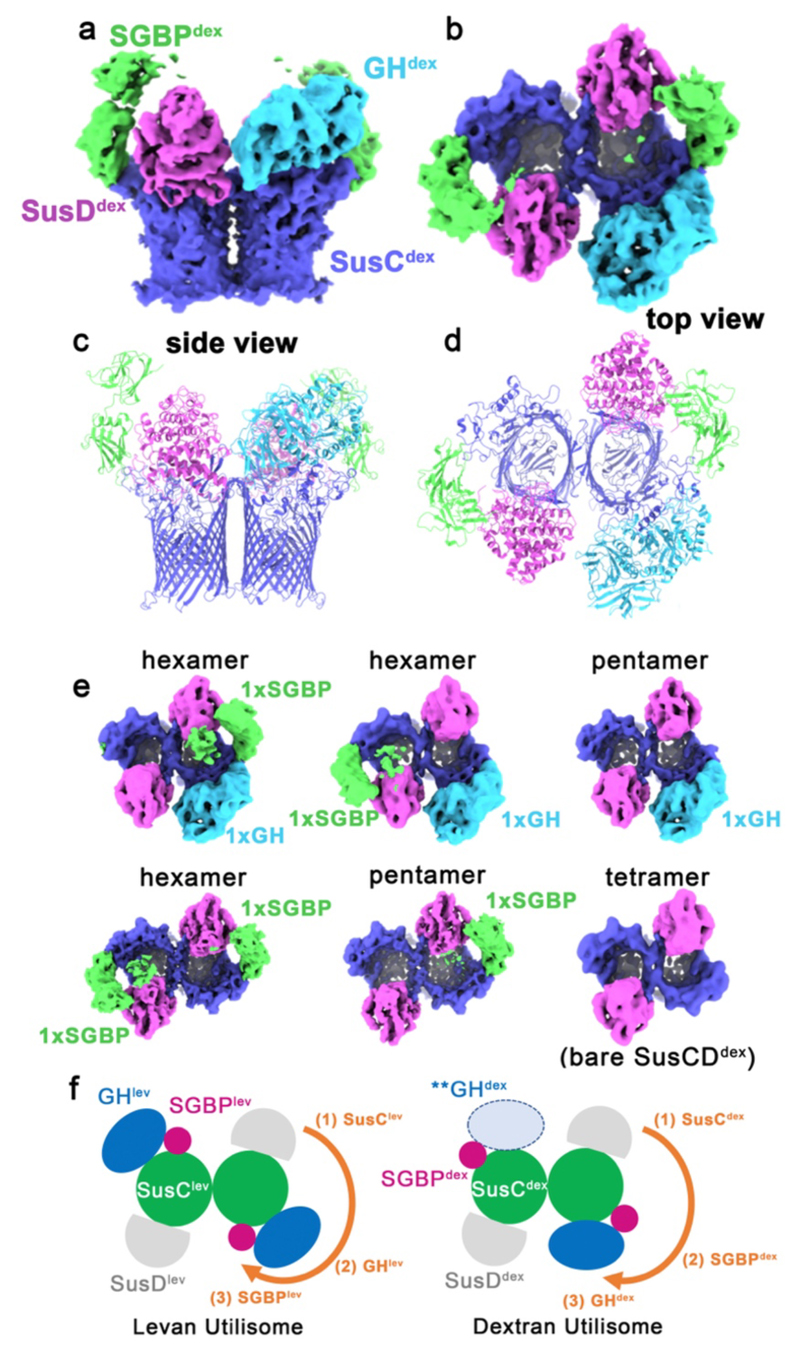
CryoEM structure and heterogeneity of the dextran utilisome observed by cryo-EM. Side (**a**) and top (**b**) view of the heptameric dextran utilisome map. The identical side (**c**) and top (**d**) views of a composite atomic model for dextran utilisome is shown. CryoEM data permitted refinement of SusC^dex^. AlphaFold2 structure predictions for SusD^dex^ and GH^dex^ were docked into the cryoEM map for the heptameric complex. An AlphaFold2 structure prediction for part of SGBP^dex^ was also fit to the cryoEM map. Unambiguous density was visible only for the first two domains of SGBP^dex^, and the predicted model was truncated prior to the C-terminal domain. SusC^dex^=purple, SusD^dex^=pink, GH^dex^=cyan and SGBP^dex^=green. The refinement for the heptameric complex had a global resolution of 3.1 Å. (**e**) Refined outputs of 3D classification viewed where each map corresponds to a unique complement or arrangement of auxiliary components (as labelled). (**f**) Schematic of the architecture for two apo glycan utilisomes. The levan utilisome (left) is coloured as in the main text (SusC^lev^=green, SusD^lev^=gray, GH^lev^=blue, and SGBP^lev^=magenta). The equivalent schematic for the substrate-free dextran utilisome is on the right. Note the different arrangement of the GH and SGBP components relative to SusD in the levan and dextran systems.

We next studied the dextran utilisation system of *B. theta*, which, like the levan PUL, encodes 4 OM components (ED Fig. 1b). Initial biochemical characterisation indicated that a utilisome existed (Fig. 1a,b), but cryoEM of the dextran complex revealed it to be much more aggregation prone than the levan utilisome (Methods). Despite this, initial 3D classification revealed that the SusCD^dex^ complex is intact, (i.e. a SusC_2_D_2_ tetramer), and that the SusD^dex^ lids are open. The positions of GH^dex^ and SGBP^dex^ were initially assigned by rigid-body docking of AlphaFold2-predicted models into additional density decorating the lip of SusC^dex^. However, unlike in the levan system, no classes corresponding to an octameric dextran utilisome existed, which we attribute to a higher propensity of the dextran utilisomes to disassemble/aggregate, potentially at the air-water interface during cryoEM grid preparation. We did observe heptameric complexes, containing two copies of the SGBP^dex^ and a single GH^dex^ (ED Fig. 6a-d), together with hexamers and pentamers encompassing all possible complements of GH^dex^ and SGBP^dex^ (ED Fig. 6e). Strikingly, although a dextran utilisome is observed, the details of its realisation in 3D are different to the levan utilisome (ED Figs. 5 and 6f). For levan, GH^lev^ is nearest to the hinge of SusD^lev^, whereas for dextran, the SGBP^dex^ is nearest to SusD^dex^. Despite this variation, for two distinct *Bacteroides* PULs these data show that all OM components required for utilisation of a glycan are assembled into a utilisome.

### Substrate binding drives conformational change in the levan utilisome

**Figure 3:**
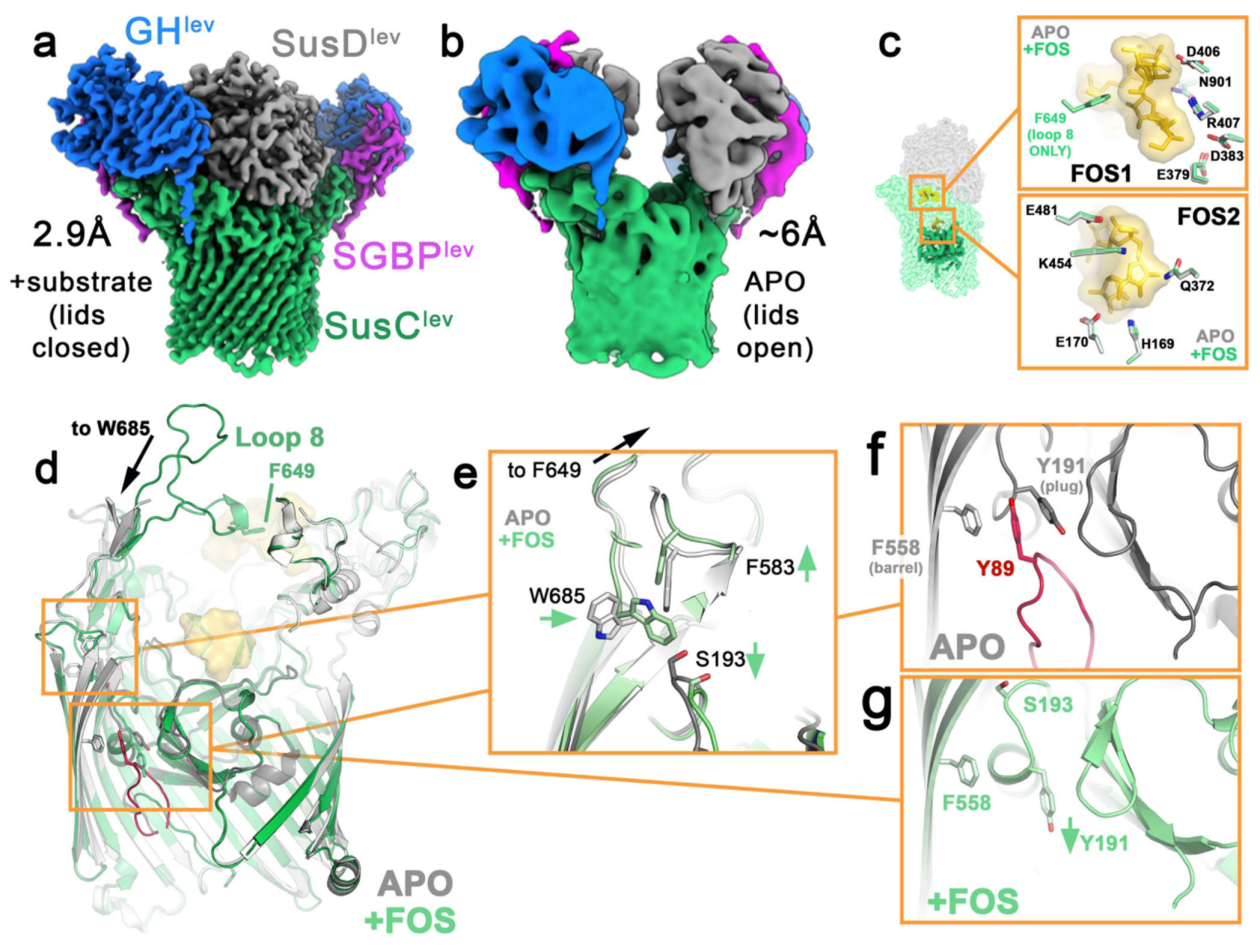
Conformational change in the utilisome upon FOS binding. (**a**) CryoEM structure of the levan utilisome with short FOS (DP8-12) at 2.9 Å resolution. (**b**) Equivalent view of the apo utilisome at 6Å resolution, showing that the SusD^lev^ lids are open in the absence of substrate and closed when it is bound. (**c**) Details of the FOS1 and FOS2 binding sites with SusC^lev^ side chains highlighted (apo in grey, FOS-bound in green) and FOS coloured yellow. F649 at the tip of loop 8 is disordered in apo, but becomes ordered in the FOS-bound utilisome. (**d**) Overview of the SusC^lev^ structure, showing loop 8, and coloured as in (**c**). The apo structure is overlaid in grey. N-terminal residues visible only in the absence of substrate are coloured red. (**e**) Zoomed-in view of residues at the base of loop 8. (**f**) The ‘aromatic lock’ in apo SusC^lev^, with Y89 in the segment that immediately precedes the TonB box (D82-G88) sandwiched between the wall of the channel (F588) and the plug (Y191). (**g**) Substrate binding disrupts the aromatic lock, and the sequence preceding the TonB box is released into the periplasm. Green arrows indicate movements in the FOS-bound structure relative to apo.

To probe substrate capture and processing by the levan utilisome, ~0.5 mM levan FOS with a degree of polymerisation of ~8-12 (DP8-12) was added to the detergent-purified utilisome complex, before performing cryoEM. The resulting 3D structure reveals that FOS binding drives major conformational change in the utilisome (Fig. 3). All SusD^lev^ lids are closed, tightly capping the extracellular face of the SusC^lev^ barrels (Fig. 3a), relative to the open position in the apo utilisomes (Fig. 3b). 3D classification reveals that the major source of heterogeneity present in the data was the presence of sub-complexes missing one or more accessory lipoproteins (ED Fig. 3b), so to probe substrate binding within the closed transporter complex, we first performed targeted refinement of the SusCD^lev^ core, using particle subtraction to remove the signal for GH^lev^ and SGBP^lev^ from the images. This allowed inclusion of all particles in the refinement, regardless of their lipoprotein complement, and yielded a reconstruction of SusCD^lev^ at 2.9 Å resolution that contains unambiguous density for FOS at two sites within the SusC^lev^ barrels (Fig. 3c). FOS1 is found at the interface between SusC^lev^ and SusD^lev^, and consists of six β2,6-linked fructose units with a β2,1 decoration on fructose-4 (numbered from the non-reducing end). This levan chain adopts a compact, twisted topology similar to that observed in the SusCD crystal structure (6ZAZ)^12^. A second binding site (FOS2) most likely consists of four β2,6-linked fructose units, and is bound at the bottom of the SusC^lev^ cavity where it contacts the top of the plug domain.

Given the utilisome’s biological role, substrate loading of the transporter should be signalled across the OM, presumably via disordering of an N-terminal region of SusC (and all TonB-dependent transporters) called the ‘TonB box’, at the periplasmic face of the complex^12^. An interesting difference between the apo and substrate-bound structures is in the position of one of the hinge points between SusC^lev^ and SusD^lev^. In the apo structure, this region (SusC^lev^ loop 8) is mostly unresolved, likely as an artefact of masking during image processing (Methods), whereas in the substrate-bound complex SusC^lev^ loop 8 contacts SusD^lev^ and FOS1 via F649 (Fig. 3c,d). However, the part of loop 8 proximal to the SusC barrel is resolved in both the apo and substrate-bound structures (Fig. 3d), with subtle changes in the side chains of its amino acid residues along the edge of the barrel and plug towards the periplasm. In the substrate-bound structure, W685, which sits at the base of loop 8, shifts inwards, nudging F583 up and S193 down (Fig. 3e). This in turn results in a downward shift of a plug loop containing S193. In the apo structure, Y191, which is part of the same plug loop, forms a triple aromatic stack with Y89 and F558 that links the wall of the barrel (F558), the TonB box (Y89) and the plug domain (Y191) (Fig. 3f). When the plug loop with Y191 shifts towards the periplasm, this stacking interaction is broken, resulting in release of the N-terminus and an ~35 Å shift of R93 (Fig. 3g), which in the substrate-bound complex is the first visible residue. This would plausibly make the TonB box (D82-G88) available for interaction with TonB to disrupt the plug and generate a channel for substrate diffusion into the periplasmic space (Supplementary Discussion). The aromatic lock in SusC^lev^ is reminiscent of *E. coli* BtuB (the TBDT for vitamin B12), where an ‘ionic lock’^25^ is broken upon vitamin B12 binding^26^ to signal extracellular substrate binding to the periplasmic face of the transporter.

### Longer substrate chains tether the levan SGBP

**Figure 4:**
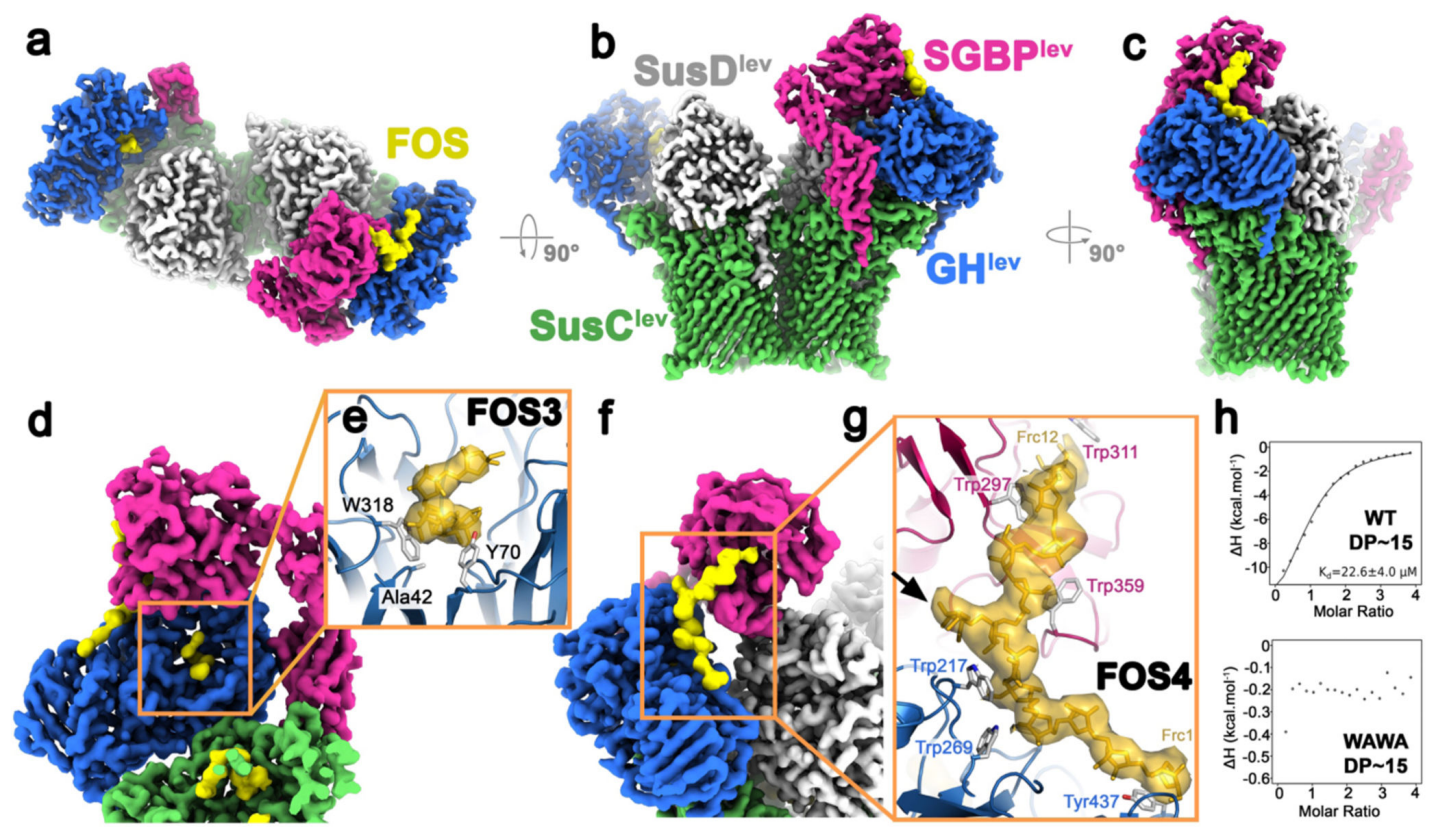
Cryo-EM structure of the holo utilisome with long FOS. Reconstruction of the levan utilisome complex after focused classification viewed (**a**) perpendicular to the plane of the membrane (from outside the cell), (**b**) rotated 90° for a side view from the OM plane, and (**c**) rotated a further 90° to give an ‘end’ view. Subunits are coloured as indicated. One SGBP^lev^ is now fully resolved (magenta), docked to the adjacent GH^lev^ subunit. (**d**) The ‘active site’ of the inactive GH^lev^ (D42A) with density for bound FOS at site 3 indicated in yellow (within the orange box). (**e**) Zoomed inset of the boxed region in (**d**), but now as a cartoon. The modelled levan chain is yellow, with aromatic side chains shown. (**f**). Zoomed view of the FOS density in the 4^th^ (FOS4) binding site that bridges the SGBP^lev^ and GH^lev^ subunits. (**g**) Proximal aromatic residues, especially tryptophans are shown (CPK coloured) and coloured according to the subunit to which they belong. The black arrow indicates the position of the putative β2,1 decoration on Frc-7. Maps displayed here have been filtered using LAFTER^64^ and segmented in UCSF Chimera. (**h**) Isothermal titration calorimetry experiments with 1 mM FOS DP~15 titrated into 50 μM recombinant wild type SGBP^lev^, or the W297A/W359A double-mutant (WAWA). Removing both tryptophan residues abolishes FOS binding.

While concerted behaviour of the levan utilisome components is implied by their arrangement, the structure of the complete levan binding protein remained unresolved. We reasoned that in a utilisome containing a catalytically inactive GH^lev^ (with the active site D42A mutation), and longer FOS chains (~DP 15-25), the binding of a single levan chain by both GH^lev^ and SGBP^lev^ might reduce conformational variability. We therefore collected such a "long-FOS" cryoEM dataset, and observed in 3D classification that a novel conformation was present, in which the C-terminal domain of SGBP^lev^ adopted a ‘docked’ state proximal to both SusD^lev^ and GH^lev^ (ED Fig. 3c.). Via focused classification without particle alignment (Methods), and using a mask including only the docked SGBP^lev^ position, we obtained a class (29k particles) containing well-resolved density for the entire SGBP^lev^ (ED Fig. 3d-f). The unmasked versions of those 29k particles were then used to generate a final reconstruction of the complete utilisome at 3.0 Å with well-resolved density for a complete SGBP^lev^ (Fig. 4a-c) (see Methods). SGBP^lev^ is organised into three domains, as predicted by Alphafold. The lipidated N-terminal domain and the central domain each have an Ig-like fold. The C-terminal levan-binding domain is globular, with a central β-sheet decorated with α-helices. A distance-matrix alignment (DALI) analysis of the C-terminal domain showed significant similarity to a ThuA-like protein (PDB ID 1T0B) with a putative role in disaccharide binding and a homoserine O-succinyl transferase (PDB ID 7CBE) (Methods).

**Extended Data Figure 7:**
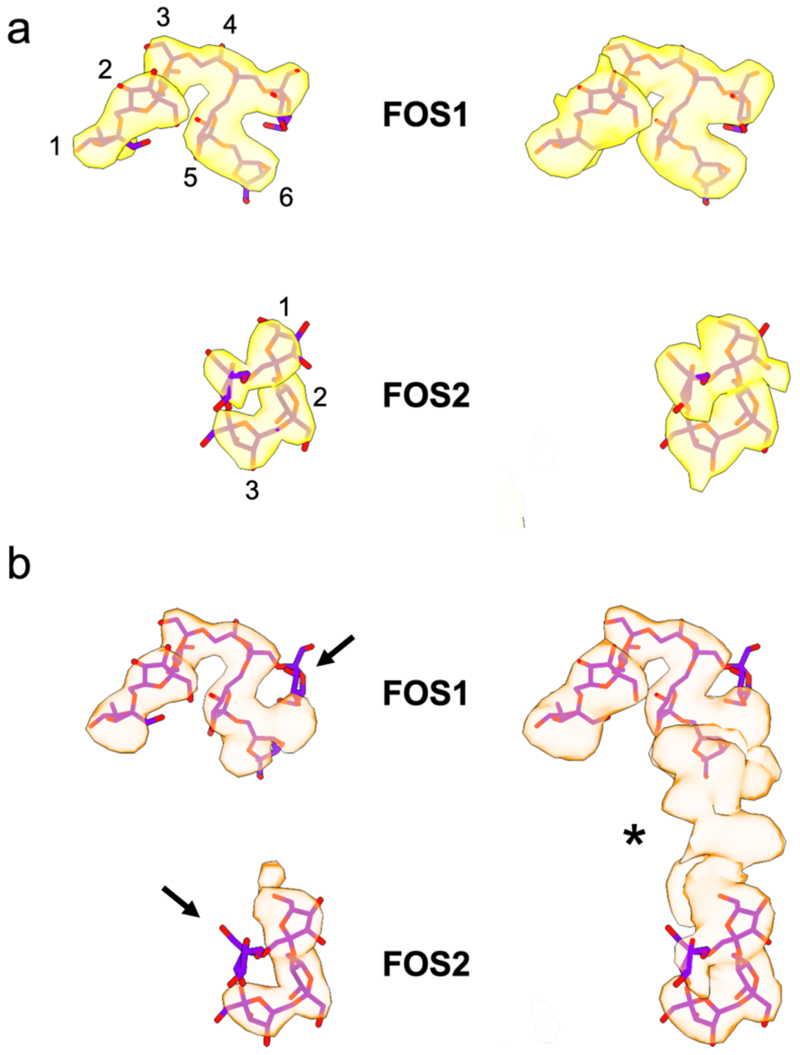
FOS binding by the SusCD^lev^ core is promiscuous. (a) Isolated FOS density obtained from the levan utilisome dataset with active GH^lev^ and short FOS (DP8-12)^12^. Density for substrate (yellow) is shown at high (left) and low (right) thresholds. (b) Isolated FOS density obtained from the utilisome structure with inactive GH^lev^ and long FOS (DP15-25). Levan density (orange) is shown at high (left) and low (right) thresholds. Arrows indicate missing fructose branches relative to (**a**). At the FOS1 site, density for the putative β2,1 decoration on Frc4 is missing. Conversely, contiguous density extends beyond the previously resolved density at FOS2, with a novel β2,1 decoration on Frc5. The substrate bound at the FOS2 site follows a similar trend with the previously modelled β2,1 linked fructose side chain being much weaker with longer FOS, while additional density attributed to another β2,6 linked monomer extends the chain towards the FOS1 site. At higher threshold levels, density connects the FOS1 and FOS2 binding sites, indicating that longer FOS (~DP15) can occupy both sites simultaneously. The connecting density is weak and indicative of multiple conformations, consistent with the absence of any contacts from SusC^lev^ to this segment. These data confirm that the transporter has considerable substrate binding promiscuity and that, as suggested previously, relatively long FOS (~15 DP) can be accommodated^12^. FOS models shown are from the original X-ray crystal structure of the SusCD^lev^ complex determined in the presence of short FOS (DP6-12)^12^.

**Extended Data Figure 8:**
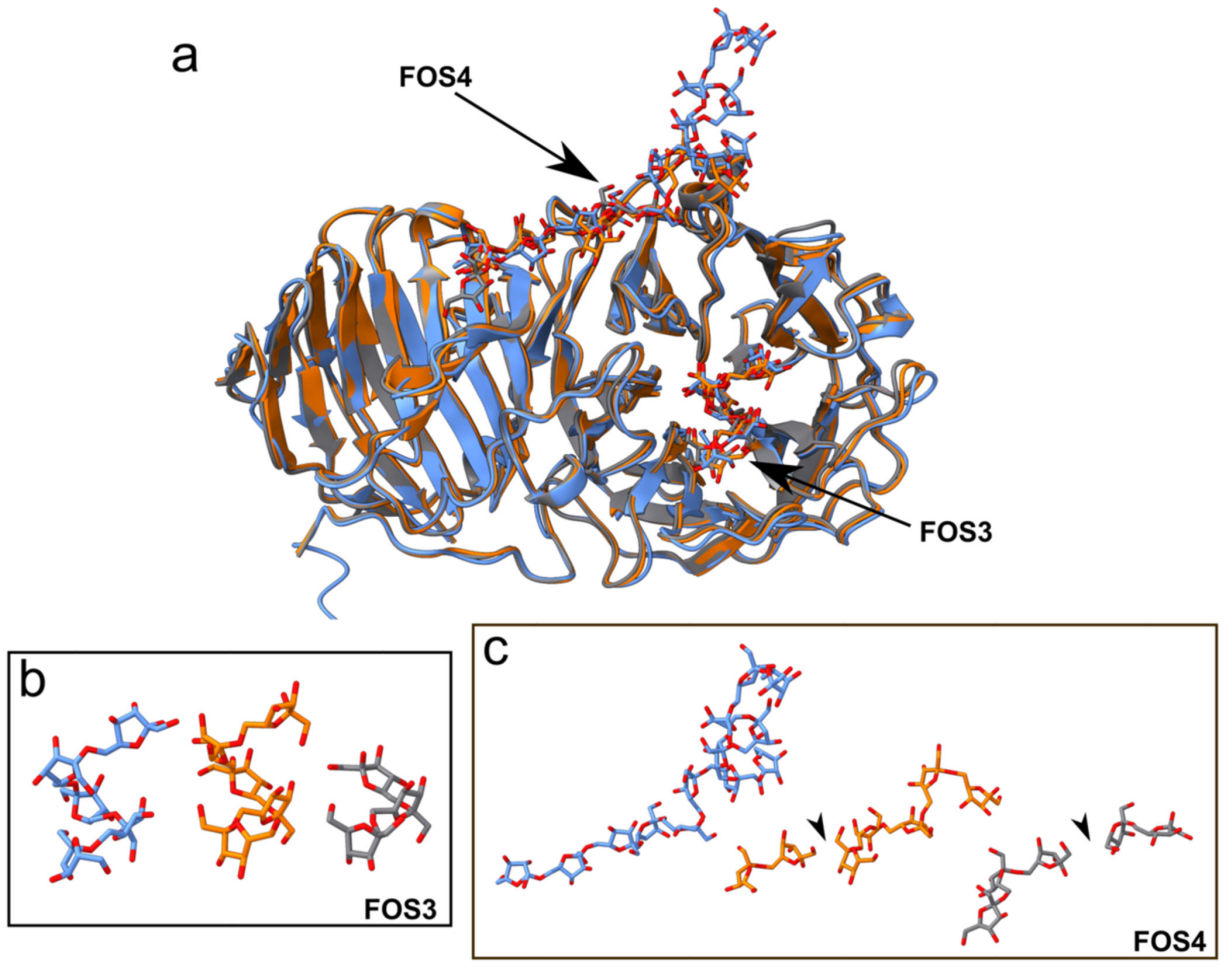
GH^lev^ structures determined by cryo-EM and X-ray crystallography reveal similar FOS binding sites. **(a)** Cryo-EM structure of the inactive GH^lev^ with FOS bound (blue) superposed with the two crystal structures (7ZNR and 7ZNS; orange, grey). (**b, c)** Comparison of FOS bound in the FOS3 (the active site) and FOS4 (secondary) binding sites of GH^lev^. The arrowheads point to breaks in the FOS chain in the crystal structures, possibly as a result of using a lower DP FOS for co-crystallization than for cryo-EM. Views in (**b)** and **(c)** are generated from a superposition.

CryoEM density, which we attribute to a length of levan chain, is found at the two locations previously described in the short FOS structure (FOS1 and FOS2). Interestingly, the substrate densities within the SusCD^lev^ binding cavity differ from those observed previously for short FOS (ED Fig. 7), suggesting that FOS binding by the SusCD^lev^ core is promiscuous. In addition, density is present at two additional sites that are unique to this long FOS structure. Within the inactivated GH^lev^ active site, there is density for ~five β2,6-linked fructose units, and we define this site as FOS3. A secondary or ‘tethering’ site of FOS binding is also observed (FOS4) with density for FOS bridging between SGBP^lev^ and GH^lev^ (Fig. 4f,g). No obvious density links the FOS3 and FOS4 binding sites. The long, bridging levan molecule binds to GH^lev^ across the top of the GH^lev^ β-propeller domain, before proceeding towards its C-terminal β-sandwich domain. The density is compatible with ~12 β2,6-linked fructose units in an extended conformation, with a putative β2,1 decoration on Frc-7 (Fig. 4g.). Levan binding at this tethering site is also seen in GH^lev^ in the absence of bridging interactions with SGBP^lev^ and in crystal structures of the inactive levanase solved in the presence of FOS of DP ~7-8 (ED Fig. 8). Indeed, secondary binding sites are relatively common in endo-acting GH enzymes, where they may enhance substrate processivity^27^. No ligand was observed at the secondary site in a GH^lev^ structure complexed to a 4 unit FOS (PDB ID 6R3U^28^), suggesting the affinity for short FOS is low.

**Extended Data Figure 9:**
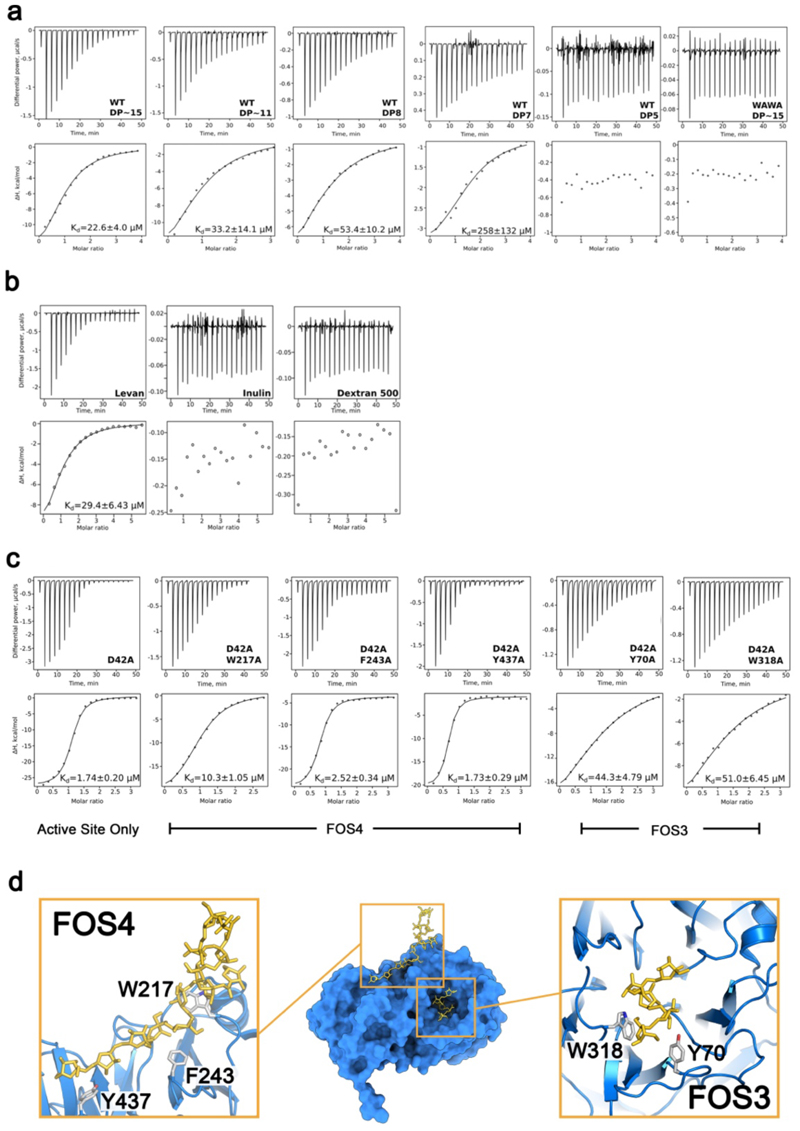
ITC of glycan binding to recombinant SGBP^lev^and GH^lev^. (a) Titration of 1 mM defined-length FOS into 50 μM wild type SGBP^lev^, suggests that ~15 fructose units are required for full affinity, which is abolished by the WAWA (W297A/W359A) mutation. (b) ITC titrations of 8 mg/ml levan, inulin or dextran 500 into 50 μM SGBP^lev^ shows its specificity for levan. (c) ITC data from titrations of GH^lev^ variants (all indicated residues mutated to alanine in the inactive D42A GH^lev^ background). Levan (8 mg/ml) was titrated into 50 μM of indicated GH^lev^ variant. Data fitting assumptions are described in the methods. (d) Surface representation of the GH^lev^ model, with FOS shown as yellow sticks. Inset are zoomed views of the FOS3 (active site) and FOS4 (secondary) binding sites, in which atomic models in cartoon representation for FOS3 are shown with side chains for aromatic residues (Y70A, W318A). For the secondary binding site these residues are W217A, F243A, Y437A.

**Extended Data Figure 10:**
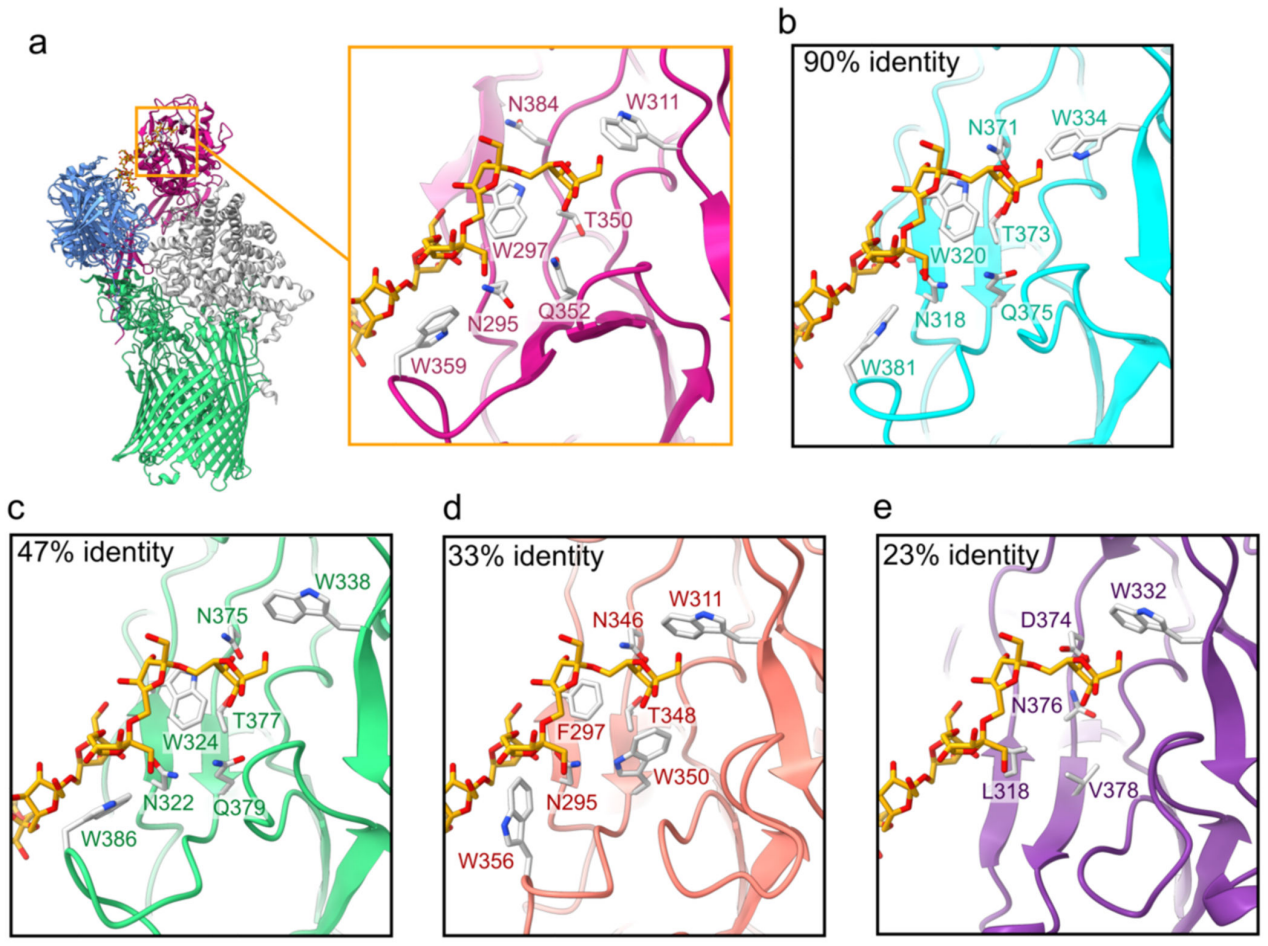
Conservation of the SGBP^lev^ β2,6-FOS binding site. **a**, Close up view of the SGBP^lev^ FOS binding site. The aromatic and polar residues that likely interact with the FOS are shown as grey stick models. The cryo-EM structure of SGBP^lev^ was aligned with selected homologue AlphaFold2-predicted models (**b**-**e**). **b**, *Bacteroides* sp. D2 SGBP^lev^ (UniProt E5CCB3). **c**, *Prevotella oralis* ATCC 33269 SGBP^lev^ (E7RM14). **d**, *Flavobacterium commune* SGBP^lev^ (A0A1D9P8I4). **e**, *F. cellulosilyticum* SGBP^lev^ (A0A4R5CJN9). FOS-binding residues equivalent to those in **a** are shown as grey stick models (if present). The FOS chain from the SGBP^lev^ cryo-EM model is shown in **b-e** for reference (orange and red). The identity indicated in each panel corresponds only to the C-terminal levan-binding domain sequence compared to SGBP^lev^ from *B. theta*. Although we could not confidently identify which SGBP^lev^ residues form hydrogen bonds with FOS from the cryo-EM maps, binding site conservation analysis indicates that N295, T350, Q352 and N384 of SGBP^lev^ are likely involved in FOS binding. The amino acid sequence alignment of the models shown here can be found in Supplementary Figure 2.

Whilst the resolution is in the region of the bridging levan is insufficient for a detailed description of binding such as hydrogen bonds (3.5-4.0 Å), several tryptophan residues are clearly involved, including W297 and W359 from SGBP^lev^, and W217 from GH^lev^, which appear to cradle the levan chain via stacking interactions. Isothermal titration calorimetry (ITC) on recombinant SGBP^lev^ confirmed the importance of W297 and W359 of the SGBP^lev^ for levan binding (Fig. 4h), and suggested that SGBP^lev^ has a single binding site, and a minimum binding unit of ~8 fructose units (ED Fig. 9a). The importance of specific stacking interactions by tryptophan residues for glycan binding platforms is consistent with previous data^29,30^ and is supported by their conservation in levan binding proteins (ED Fig. 10). Indeed, ITC indicates that SGBP^lev^ has little affinity for inulin, a fructose oligosaccharide similar to levan but with different main chain linkages (β2,1 instead of β2,6) (ED Fig. 9b), confirming the specificity of binding. We next attempted to dissect the affinities of the FOS3 and FOS4 binding sites, both of which include numerous aromatic residues (ED Fig. 9c,d). Single substitution of aromatic residues (to alanine) near the GH^lev^ active site (Y70A and W318A) decreased affinity for levan 25-30 fold, while equivalent substitutions at the tethering site FOS4 had a 6-fold decrease in affinity (W217A) or no effect (F243A and Y437A). These results suggest the active site (FOS3) is responsible for most of the affinity of GH^lev^ for levan. However, numerous weak interactions within the extensive secondary site (FOS4) including polar residues, may result in a substantial overall affinity and specificity.

The utilisome structure suggests why SusCD transporters exist as dimers. The SGBP^lev^ is clearly inherently flexible, and in the dataset with inactive GH^lev^ and long FOS, a novel conformation was observed in which an untethered SGBP^lev^ from one SusC^lev^ contacts the tethered SGBP^lev^ associated with the other SusC^lev^ (ED Fig. 4e). This conformation could result in both SGBP^lev^ interacting with the same stretch of a long levan chain (as would be present *in vivo)*, increasing substrate avidity and helping to retain and position it near the utilisome for processing and transport.

## Discussion

**Figure 5:**
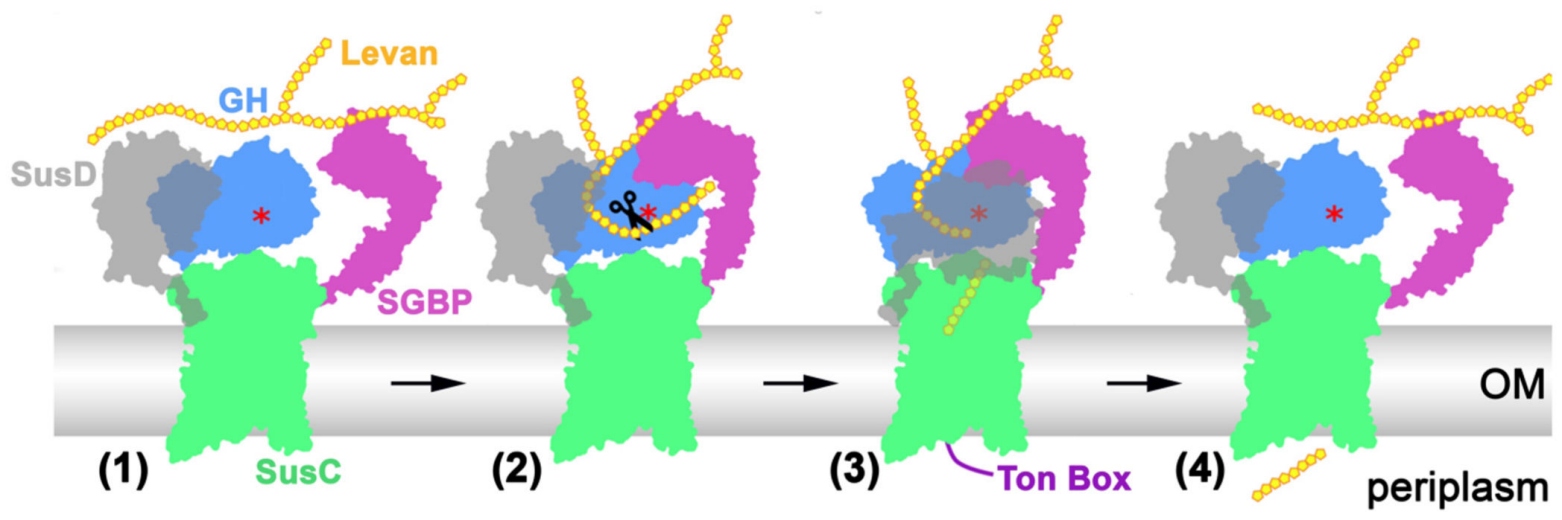
The transport pathway of utilisomes dedicated to the processing and import of simple glycans. Description of states (1-4) is provided in the main text. N.B. only one half of the dimeric utilisome is shown for clarity. A red asterisk marks the active site of the glycoside hydrolase (GH). OM=outer membrane.

Our utilisome cryo-EM structures provide new insight into glycan acquisition by gut Bacteroidetes. As Bacteroidetes are found in a diverse range of terrestrial and marine niches^31^, and have all been shown to encode SusCD homologues^32^, the data may also expand our understanding of glycan utilisation outside the animal gut. We show that for two PULs dedicated to the breakdown of simple glycans, all of the gene products that localise to the OM form a stable utilisome complex. The way in which these utilisomes are realised in 3D are subtly different (ED Fig. 6f), but they have closely-related architectures that rationalise function (Fig. 5). In the absence of substrate (1), the glycan binding domain of an SGBP is mobile, increasing its efficiency as a glycan grappling device. Likewise, the SusD lids, which can open and close without clashing with the other components, are mobile but open. (2) Glycan binding by the SGBP is followed by docking to the proximal GH, and the glycan binds to both the tethering and active sites of the enzyme. Substrate cleavage by GH then generates shorter oligosaccharides close to the mouth of the SusCD transporter. (3) Binding of the oligosaccharide to SusCD promotes lid closure and signalling to the plug, breaks the aromatic lock and exposes the Ton box to the periplasm. This is followed by TonB-dependent transport events (Supplementary Discussion), and resetting of the transporter to the open state (4)^10,12^.

The long levan chain between SGBP^lev^ and GH^lev^ is much more extended than at other sites, and may therefore represent a ‘strained’ state in which a bound levan chain is pulled taut. However, we note that this conformation represents the least mobile and therefore most resolvable state, and has thus been ‘selected for’ in cryoEM image processing. Furthermore, although the GH^lev^ tethering site (FOS4) is ~25 Å away from its catalytic site (FOS3), we speculate that the SGBP:GH interaction increases the levan concentration local to the active site, potentially enhancing the efficiency of substrate cleavage. Flexibility of the levan chain away from the interaction sites may preclude resolution of contiguous density in our structure, or the FOS used here may be too short to bridge both sites.

Whilst our data argue for stable utilisome assemblies in the OM, recent studies on the starch PUL propose a more dynamic model, with two SGBPs forming immobile starch binding centres, around which a GH and transporter components transiently assemble^21^. Proteomics data for the starch utilisation system reveal similar amounts of SusC and SusD at the OM whilst SusEFG are much less abundant, suggesting the presence of complexes with differing stoichiometries^21^. Intriguingly, co-immunoprecipitation with SusD antibodies captures twice as much SusD as SusC, suggesting that even the core SusCD transporter may not form a stable complex in the starch system. This contrasts with our work, where separate SusC or SusD components have not been observed. Moreover, while it is not clear how similar the outer membranes of *E. coli* and *B. theta* are, recent work has shown that *E. coli* OMP and LPS mobility is limited ^33,34^, raising questions about the efficiency of a complex that dynamically (dis)assembles in a crowded OM. It is not clear why the system for starch appears to operate differently to those for levan and dextran. The dynamic model for the starch PUL is based on live-cell fluorescence studies with C-terminal fusions of OM PUL components with relatively large fluorescent protein tags, which may destabilise the utilisomes but not markedly affect cell growth *in vitro*. On the other hand, the starch PUL is different as it encodes two SGBPs (SusE and SusF), *i.e*. there are five OM PUL components and not four as in the systems studied here. An AlphaFold2 structure prediction of the starch SusC (Bt3702) shows that it has no extra interaction surface for the association of additional lipoprotein components relative to the levan and dextran SusCs, making it unlikely that SusE, SusF and SusG can all bind the same SusCD transporter and suggesting the starch utilisome may be more dynamic, and/or perhaps forms distinct, specialised utilisomes.

Indeed, the diversity of known PULs is considerable. Determining whether four-component utilisomes, as presented here, are a generic feature of PULs will require the study of more systems from gut *Bacteroides spp*. (both *B. theta* and others), as well as from more distant Bacteroidetes occupying different ecological niches such as soil and marine environments. The levan and dextran utilisation systems described here target relatively simple glycans, and both the number of PULs and their compositional complexity appears to correlate with the complexity of their substrates, *i.e*. the number of encoded SGBPs, carbohydrate active enzymes, and even SusCD pairs increases in-line with substrate complexity^35–37^. Whether the components of these complex PULs can undergo mix-and-match-style assembly, resulting in the formation of several utilisome complexes with unique lipoprotein complements is unknown, but represents an important target of future study.

## Methods

### Construction of *B. theta* strains

*B. theta* strains were made as described previously ^12^. Briefly, the DNA sequence containing the desired alterations was constructed using the sewing PCR method and ligated into the pExchange-tdk vector^38^. The vectors carrying the altered DNA sequences were introduced into the *B. theta tdk*^*-*^ strain via conjugation from *E. coli* S17 λ *pir*. Chromosomal alterations were made by allelic exchange, followed by selection for loss of the pExchange-tdk vector backbone. Mutations were confirmed by PCR amplification of the region of interest and Sanger sequencing.

### Expression and purification of utilisomes from *B. theta*

*B. theta* strains were grown at 37°C in a Don Whitley Scientific A32 anaerobic workstation. Brain-heart infusion (BHI) cultures supplemented with 2 ug/ml hemin were inoculated with stabs from glycerol stocks of the appropriate *B. theta* strain and incubated overnight. Defined minimal medium^12^ was supplemented with 2 ug/ml hemin and either 0.5% fructose (levan system) or 0.5% dextran 3.5 kDa (dextran system) and inoculated with the overnight BHI cultures (1:1000 dilution). The cultures were harvested after 20 h by centrifugation and the pellets were stored at -20°C. 4 litres of culture were grown for SusC^lev^-His (with wild type GH^lev^) and SGBP^lev^-His/GH^lev^-D42A (inactive GH^lev^) strains for cryo-EM.

The cell pellets were thawed, supplemented with DNase I and homogenised in Tris-buffered saline (TBS, 20 mM Tris-HCl pH 8.0, 300 mM NaCl). The cells were lysed with a single pass at 22 kpsi through a cell disruptor (0.75 kW; Constant Systems). Membranes were isolated by ultracentrifugation for 45 min at 42,000 rpm (45 Ti rotor, Beckman), 4°C. The membranes were solubilised at 4°C for 1 h in TBS with 1% DDM while stirring. Insoluble material was pelleted by ultracentrifugation for 30 min at 42,000 rpm (45 Ti rotor) at 4°C. The supernatants were supplemented with 20 mM imidazole and loaded onto an 8 ml chelating sepharose column charged with Ni^2+^, by gravity flow at room temperature. The column was washed with 20 column volumes TBS with 30 mM imidazole and 0.15% DDM. The bound proteins were eluted with 3 column volumes TBS with 250 mM imidazole and 0.15% DDM. The eluates were concentrated in an Amicon Ultra filtration device with a 100 kDa cut-off membrane. Levan utilisome samples were then loaded on a HiLoad 16/600 Superdex 200 pg column (Cytiva) in 10 mM HEPES-NaOH pH 7.5, 100 mM NaCl, 0.03% DDM. For dextran utilisomes, SEC was carried out using Superose 6 columns, given that dextran binding proteins absorb to Superdex 200 resin. The separation profiles of both column types are different, explaining the different elution times for the similarly-sized complexes in Fig. 1b. Fractions containing pure protein were pooled, concentrated, flash-frozen in liquid nitrogen and stored at -80°C.

### Sample preparation for mass spectrometry

*B. theta* cells were grown in triplicate in minimal medium with 0.5% fructose or 0.5% dextran 3.5 kDa, harvested by centrifugation and lysed as above. Total membrane pellets were extracted twice with 0.5% sarkosyl in 20 mM Hepes pH 7.5 (20 mins at room temperature) to remove inner membrane components. Each extraction was followed by centrifugation for 30 min at 42,000 rpm (45 Ti rotor), and the pellet was retained. OM samples were suspended in 5% sodium dodecyl sulfate (SDS) in 50 mM triethylammonium bicarbonate (TEAB) pH 7.5 and sonicated using an ultrasonic homogenizer (Hielscher) for 1 minute. Samples were centrifuged at 10,000 xg for 10 minutes to pellet debris. Proteins (40 μg) were subsequently reduced by incubation with 20 mM tris(2-carboxyethyl)phosphine for 15 minutes at 47 °C, and alkylated with 20 mM iodoacetamide for 15 minutes at room temperature in the dark. Proteomic sample preparation was performed using the suspension trapping (S-Trap) sample preparation method^39,40^, with minor modifications as recommended by the supplier (ProtiFi™, Huntington NY). Briefly, 2.5 μl of 12% phosphoric acid was added to each sample, followed by the addition of 165 μl S-Trap binding buffer (90% methanol in 100 mM TEAB pH 7.1). The acidified samples were added, separately, to S-Trap micro-spin columns and centrifuged at 4,000 xg for 1 minute until all the solution has passed through the filter. Each S-Trap micro-spin column was washed with 150 μl S-trap binding buffer by centrifugation at 4,000 xg for 1 minute. This process was repeated for a total of five washes. Twenty-five μl of 50 mM TEAB containing 4 μg trypsin was added to each sample, followed by proteolytic digestion for 2 hours at 47 °C using a thermomixer (Eppendorf). Peptides were eluted with 50 mM TEAB pH 8.0 and centrifugation at 3,000 xg for 1 minute. Elution steps were repeated using 0.2% formic acid and 0.2% formic acid in 50% acetonitrile, respectively. The three eluates from each sample were combined and dried using a speed-vac before storage at -80°C.

### Mass spectrometry

Peptides were dissolved in 2% acetonitrile containing 0.1% formic acid, and each sample was independently analysed on an Orbitrap Q Exactive HF mass spectrometer (Thermo Fisher Scientific), connected to an UltiMate 3000 RSLCnano System (Thermo Fisher Scientific). Peptides were injected on a PepMap 100 C18 LC trap column (300 μm ID × 5 mm, 5 μm, 100 Å) followed by separation on an EASY-Spray nanoLC C18 column (75 μm ID × 50 cm, 2 μm, 100 Å) at a flow rate of 250 nl/min. Solvent A was water containing 0.1% formic acid, and solvent B was 80% acetonitrile containing 0.1% formic acid. The gradient used for analysis was as follows: solvent B was maintained at 2% B for 5 min, followed by an increase from 2 to 30% B in 110 min, 30% to 42% B in 10 min, 42-90% B in 0.5 min, maintained at 90% B for 4 min, followed by a decrease to 2% in 0.5 min, and equilibration at 2% for 20 min. The Orbitrap Q Exactive HF was operated in positive-ion data-dependent mode. The precursor ion scan (full scan) was performed in the Orbitrap (OT) in the range of 350-1,500 m/z with a resolution of 60,000 at 200 m/z, an automatic gain control (AGC) target of 3 × 10^6^, and an ion injection time of 50 ms. MS/MS spectra were acquired in the OT using the Top 20 precursors fragmented by high-energy collisional dissociation (HCD) fragmentation. Precursors were isolated using the quadrupole using a 1.6 m/z isolation width. An HCD collision energy of 25% was used, the AGC target was set to 2 × 10^5^ and an ion injection time of 50 ms was allowed. Dynamic exclusion of ions was implemented using a 45 s exclusion duration. An electrospray voltage of 1.8 kV and capillary temperature of 280°C, with no sheath and auxiliary gas flow, was used.

### Mass spectrometry data analysis

All spectra were analysed using MaxQuant 1.6.14.0 ^41^, and searched against the *Bacteroides thetaiotaomicron* Uniprot proteome database (UP000001414) downloaded on 22 September 2020. Peak list generation was performed within MaxQuant and searches were performed using default parameters and the built-in Andromeda search engine ^42^. The enzyme specificity was set to consider fully tryptic peptides, and two missed cleavages were allowed. Oxidation of methionine and N-terminal acetylation were set as variable modifications. Carbamidomethylation of cysteine was set as a fixed modification. A protein and peptide false discovery rate (FDR) of less than 1% was employed in MaxQuant. Proteins that contained similar peptides and that could not be differentiated based on MS/MS analysis alone were grouped to satisfy the principles of parsimony. Reverse hits, contaminants, and proteins only identified by site modifications were removed before downstream analysis. Ranking of protein abundance was performed using iBAQ intensity values^22^ obtained from MaxQuant. Label-free quantification was performed using LFQ intensities obtained from MaxQuant. LFQ intensities were log2 transformed and filtered to contain at least three valid values in one of the groups. Missing values were imputed using the minProb function in the imputeLCMD package (https://cran.r-project.org/web/packages/imputeLCMD) in R version 4.1.1. Statistical analysis was performed using limma^43^ and the Benjamini-Hochberg correction for multiple hypothesis testing was implemented.

### Construction of plasmids for protein expression in *E. coli*

The nucleotide sequences encoding GH^lev^ (Bt1760; residues 2-503) and SGBP^lev^ (Bt1761; residues 2-438) were amplified from *B. theta* genomic DNA, excluding the signal sequence and the lipid anchor cysteine. In all cases, the protein numbering starts with the first residue of the mature sequence, corresponding to C21 in GH^lev^ and C24 in SGBP^lev^ precursor amino acid sequences. The PCR product encoding GH^lev^ was digested with NcoI and XhoI and ligated into pET28b, resulting in fusion of the coding sequence to a C-terminal LEHHHHHH tag. The PCR product encoding SGBP^lev^ was digested with NdeI and XhoI and ligated into pET28a, resulting in fusion of the SGBP^lev^ coding sequence to an N-terminal MGSSHHHHHHSSGLVPRGSHM tag. TOP10 cells were transformed with the ligation mixtures and plated on LB agar plates with kanamycin. After overnight incubation at 37°C, clones were screened for successful ligation by colony PCR. GH^lev^ and SGBP^lev^ variants were generated using the Q5 site directed mutagenesis kit (NEB). All constructs were verified by Sanger sequencing.

### Expression and purification of proteins from *E. coli*

GH^lev^, SGBP^lev^ and their variants were overexpressed in *E. coli*. Electrocompetent *E. coli* BL21(DE3) cells were transformed with the appropriate plasmid, plated on LB kanamycin plates and incubated at 37°C overnight. Starter LB kanamycin cultures were inoculated by scraping the transformants and incubated at 37°C, 180 rpm for 2 hours. 13 ml of the starter culture were used to inoculate each litre of LB kanamycin. The cultures were incubated at 37°C, 180 rpm until OD600 0.5-0.6 and induced with 0.2 mM IPTG. The temperature was then lowered to 20°C and the cultures were incubated for a further 19-21 h. The cells were harvested by centrifugation and the pellets were stored at -20°C.

The pellets were thawed, supplemented with DNase I and homogenised in TBS buffer. The cells were lysed with a single pass at 25 kpsi through a cell disruptor (Constant Systems). The lysates were supplemented with 1 mM PMSF. Unbroken cells were pelleted by centrifugation for 30 min at 30,000g, 4°C. The supernatants were loaded on a 5 ml Ni^2+^-charged chelating sepharose column by gravity flow at room temperature. The column was washed with 40 column volumes TBS buffer containing 30 mM imidazole, and the bound proteins were eluted with 5 column volumes TBS buffer containing 250 mM imidazole. The eluates were concentrated in an Amicon Ultra filtration device (30 kDa cut-off) by centrifugation. The samples were then loaded in batches on a HiLoad 16/600 Superdex 200 pg column (Cytiva) in 10 mM HEPES-NaOH pH 7.5, 100 mM NaCl. Elution fractions were collected and analysed by SDS-PAGE for purity. Fractions containing the proteins of interest were pooled, concentrated, flash-frozen in liquid nitrogen, and stored at -80°C.

### Fructooligosaccharide production

FOS used for cryoEM, crystallography and ITC were generated by partial digestion of *Erwinia herbicola* levan (Sigma) by GH^lev^ (Bt1760) as described previously^12^.

### Isothermal titration calorimetry

Protein samples were thawed, centrifuged to remove any aggregates, and diluted to 25 or 50 mM in 10 mM HEPES-NaOH pH 7.5, 100 mM NaCl. Levan from *E. herbicola* or defined-length FOS were dissolved in the same buffer to 8 mg/ml and 1 mM, respectively. ITC was performed using a Microcal PEAQ-ITC instrument (Malvern Panalytical). Levan or FOS was injected into the sample cell containing protein or buffer. The titrations were performed at 25°C. The sample cell was stirred at 750 rpm. After an initial delay of 60 s, an injection of 0.4 ml was done (which was discarded from data analysis) followed by 18 injections of 2 ml. The spacing between injections was 150 s. Ligand to buffer control titrations were subtracted from all experiments. The experiments were repeated at least twice. Data were fitted to a single binding site model using the Microcal PEAQ-ITC Analysis software v1.40. It was impossible to determine the precise molar concentration of the levan titrant due to heterogeneity in chain length. Therefore, the molarity of available binding sites was estimated during data fitting. For GH^lev^, the only secondary binding site substitution that had an effect on the affinity was W217A. We assumed that all the affinity observed for this variant could be attributed to binding to the active site alone. Therefore, the stoichiometry was fixed to 1 and the ligand concentration was floated during data fitting. The estimated molar concentration of 0.8% levan was 829 μM. N.B. the “molarity” in this case corresponds to the number of accessible binding sites for the enzyme on the polymeric levan ligand, rather than number of molecules, per volume. This titrant concentration was fixed for all other data fits for GH^lev^. Similarly, by fixing n to 1, the levan titrant concentration for SGBP^lev^ was determined to be 1.48 mM, suggesting ~2x the number of binding sites on levan available to the SGBP as to the enzymes active site. Notably, the affinity of SGBP^lev^ for levan determined this way was similar to that determined using defined-length FOS with known molarity (ED figure 9).

### Protein crystallisation

GH^lev^ (Bt1760_SeMet) was crystalized in the presence of 200 mM potassium/sodium tartrate, 100 mM sodium citrate pH 5.6, 1.4 M ammonium sulphate and 500 mM fructose. GH^lev^_D42N crystal forms were crystalized in 1.5 M lithium sulphate and 200 mM ammonium sulphate, 100 mM MES pH 6.5 and 30% PEG 5000 MME respectively. SGBP^lev^ (both native and SeMet protein) was crystallised using 1.8-2.2 M (NH_4_)_2_SO_4_, 0.1 M MES pH 6.5. The protein concentrations were in the range of 10 mg/ml. The drops, composed of 0.1 μl or 0.2 μl of protein solution plus 0.1 μl of reservoir solution, were set up using a Mosquito crystallization robot (SPT Labtech). The vapor diffusion sitting drop method was used and the plates were incubated at 20 °C. If required, crystal hits were optimised via hanging drop vapour diffusion with larger volume drops (typically 1-1.5 μl). GH^lev^_SeMet samples did not require additional cryoprotection. GH^lev^_D42N samples were cryoprotected with paratone-N and with addition of 20% PEG 400 to the reservoir respectively. SGBP^lev^ samples were cryoprotected by adding 4-fold excess of 3.5 M (NH_4_)_2_SO_4_ to the crystal drops.

### Data collection, structure solution, model building, refinement and validation

Diffraction data were collected at the synchrotron beamlines I02, I03 and I04 of Diamond Light Source (Didcot, UK) at a temperature of 100 K. The data set for GH^lev^ SeMet was integrated with DIALS^44^ via XIA2^45^ and scaled with Aimless^46^. The space group was confirmed with Pointless^47^. The phase problem was solved by experimental phasing with Crank2^48^. Mutant GH^lev^_D42N data sets were integrated by XDS^49^ and processed as above. After phase transfer from experimental phasing the automated model building program task CCP4build on CCP4cloud^50^ delivered models with Rfactors below 30 %. The models were refined with Refmac^51^ and manual model building with COOT^52^. The final models were validated with COOT and MolProbity. Data collection and refinement statistics are presented in Supplementary Table 1. Other software used were from CCP4 suite^53^. Data collected for SeMet SGBP^lev^ allowed solving the phase problem and partial model building via single anomalous dispersion (Se-SAD) using Phenix AUTOSOL^54^. Iterative rounds of manual building within COOT and model refinement in Phenix resulted in a partial model with R_free_ ~35%, which was used as the input for model completion in the cryo-EM maps of the levan utilisome with long FOS. The segments missing from the SGBP^lev^ X-ray model could not be modelled using the complete cryo-EM structure due to the poor quality of the X-ray electron density maps in the missing regions.

### Levan utilisome cryo-EM sample preparation, data collection and image processing

A sample of the purified apo levan utilisome complex solubilised in DDM-containing buffer (10 mM HEPES, pH 7.5, 100 mM NaCl, 0.03% DDM) was prepared at 3 mg/ml. Lacy carbon 300-mesh copper grids (Agar Scientific) were glow-discharged in air (10 mA, 30s, Cressington 208). A sample volume of 3.5 mL was applied to the grid. Blotting and plunge freezing into liquid nitrogen-cooled liquid ethane were carried out using an FEI Vitrobot Mark IV (Thermo Fisher Scientific) with chamber conditions set at a temperature of 4 °C and 100% relative humidity. The grid was blotted for 6 s with a blot force of 6. Micrograph movies were collected on a Titan Krios Microscope (Thermo Fisher Scientific) operating at 300 kV with a Falcon III direct electron detector operating in counting mode. Data acquisition parameters can be found in Supplementary Table 2. Density maps coloured according to local resolution, together with angular distribution plots, are included for each EM map described in this study (Supplementary Figure 3).

An initial dataset comprising 2057 micrograph movies was collected and image processing was carried out in Relion3.1^55^. Drift correction and dose-weighting was carried out using MOTIONCOR2^56^. CTF estimation of motion corrected micrographs was performed using Gctf^57^. Template-based particle picking within Relion was hindered by the large amount of carbon present in many micrographs. The micrograph stack was therefore manually culled to remove micrographs containing >50% carbon, leaving 1093 micrographs for further processing. Final particle picking was performed using the crYOLO general model^58^, yielding 96,639 particles. This particle stack was subjected to several rounds of 2D classification, after which 89,305 particles remained. A 3D starting model was generated *de novo* from the data using the stochastic gradient descent algorithm within RELION. 3D classification was used to isolate particles corresponding to the complete octameric utilisome complex (45,594). These particles were subjected to further rounds of classification in 3D to assess the conformational heterogeneity of the complex. Classification revealed considerable heterogeneity in the position of the SusD lids, with positions described as ‘wide open’ (W) and ‘narrow open’ (N) identified in all possible combinations. WW, WN and NN states contained 16,155, 22,452 and 6,987 particles respectively. To increase particle numbers and improve the results of downstream processing a second dataset of 3142 movies was collected. These were processed in the same way as described for the initial dataset, with particles picked using crYOLO. Classification in 3D yielded 146,512 particles that corresponded to the complete octameric utilisome complex. To improve the resolution for the more static, C2 symmetric portions of the utilisome (SusC^lev^, GH^lev^ and N-terminal region of the SGBP^lev^) particles stacks corresponding to the complete octameric complex from both datasets (192,106 total) were combined and subject to focused refinement with C2 symmetry. The mask applied in focused refinement excluded the SusD subunits. Particles were subjected to iterative rounds of CTF-refinement and Bayesian polishing (run separately for each dataset) until no further improvement in resolution was seen. Post-processing was performed using a soft, extended mask and yielded a global sharpened reconstruction at 3.5 Å, as estimated by the gold standard Fourier shell correlation using the 0.143 criterion.

### Active levan utilisome in the presence of FOS (DP8-12) cryo-EM sample preparation, data collection and image processing

A sample of the levan utilisome containing an active GH^lev^ solubilised in a DDM-containing buffer (10 mM HEPES, pH 7.5, 100 mM NaCl, 0.03% DDM) was prepared at 3 mg/ml and incubated with 0.5 mM levan FOS with a degree of polymerisation of ~8-12 for at least one hour before grid preparation. Quantifoil carbon grids (R1.2/1.3, 300 mesh) were glow discharged (30 mA, 60 s, Quorum GloQube) in the presence of amylamine vapour. A sample volume of 3.5 mL was applied to the grid. Blotting and plunge-freezing into liquid nitrogen-cooled liquid ethane were carried out using an FEI Vitrobot Mark IV (Thermo Fisher Scientific) with chamber conditions set at a temperature of 4 °C and 100% relative humidity. The grid was blotted for 6 s with a blot force of 6. Micrograph movies were collected on a Titan Krios Microscope (Thermo Fisher Scientific) operating at 300 kV with a Falcon III direct electron detector operating in counting mode. Data acquisition parameters can be found in Supplementary Table 3.

A dataset comprising 974 micrograph movies was collected and image processing was carried out in RELION 3.1 ^55^. Drift correction and dose-weighting was done using MOTIONCOR2 ^56^. CTF estimation of motion corrected micrographs was performed using Gctf ^57^. Particle picking was performed using the general model of crYOLO and yielded 72,373 particles ^58^. Unwanted particles/contamination were removed from the particle stack through two rounds of 2D classification, after which 63,789 particles remained. Classification in 3D was used to address compositional heterogeneity. Good classes containing unambiguous SusC_2_D_2_ density represented the complete octameric utilisome, a hexameric assembly which lacked one GH^lev^ and one SGBP^lev^ subunit and a naked SusC_2_D_2_ complex. Contributing particles numbers were 17,045, 31,789 and 7390, respectively. SusD lids invariantly occupied a closed position and conformational heterogeneity was limited to the position of the levan binding protein.

Complete utilisome particles were subjected to iterative rounds of CTF-refinement and Bayesian polishing until no further improvement in resolution was seen. Post-processing was performed using a soft, extended mask and yielded a global sharpened reconstruction at 3.2 Å, as estimated by the gold standard Fourier shell correlation using the 0.143 criterion.

To extract higher resolution information for the SusCD^lev^ core complex, particle subtraction was performed to remove signal for additional lipoprotein components from all experimental images contributing to good classes (as defined above). A soft, expanded mask encompassing only the SusCD^lev^ core was generated using the volume eraser tool within Chimera ^59^ before using the resulting carved volume as in input for mask creation in RELION. Subtracted particles were used in a focused refinement with the same mask applied while enforcing C2 symmetry. Iterative rounds of CTF-refinement and Bayesian polishing were employed until no further improvement in resolution was observed. Post-processing resulted in a final sharpened reconstruction at 2.9 Å.

### Inactive levan utilisome in the presence of FOS (DP15-25) cryo-EM sample preparation, data collection and image processing

A sample of the levan utilisome containing inactivated GH^lev^ (D42A), solubilised in a DDM-containing buffer (10 mM HEPES, pH 7.5, 100 mM NaCl, 0.03% DDM), was prepared at 3 mg/ml and incubated with ~0.5 mM levan FOS with a degree of polymerisation 15-25 for at least one hour before grid preparation. Grid type, preparation, microscope and detector were the same as for the active levan utilisome described above. Data acquisition parameters can be found in Supplementary Table 4.

A dataset comprising 1388 micrograph movies was collected and image processing was carried out in RELION 3.1 ^55^. Drift correction and dose-weighting were carried out using MOTIONCOR2 ^56^. Particle picking was performed using the general model of crYOLO and yielded 157,953 particles^58^. Unwanted particles/contamination were removed from the particle stack through two rounds of 2D classification, after which 146,056 particles remained. Classification in 3D was used to address compositional heterogeneity. Good classes representing the complete octameric utilisome and the hexameric assembly lacking one SGBP^lev^ and one GH^lev^ subunit were observed and contained 78,469 and 42,488 particles, respectively. Conformational heterogeneity in the position of the levan binding protein was observed with some classes possessing a conformation where this subunit was held close to GH^lev^. Particles contributing to all classes with evidence of this docked conformation of the levan binding protein were pooled (98,755) and a consensus refinement was carried out. CTF refinement and Bayesian polishing were performed iteratively until no further improvement in resolution was observed. The resulting reconstruction possessed weaker density for the C-terminal domain of the levan binding protein than for the N-terminal portions. To improve this, a focused classification approach without alignment was used.

A mask encompassing only the docked position of the SGBP^lev^ with some surrounding density was created using the volume eraser tool in Chimera followed by mask creation in RELION. A focused 3D classification job without alignment was run using the aforementioned mask and the output from the aforementioned refinement as a reference model. The reference model was low-pass filtered to 3.5 Å, just above the resolution of 3.3 Å reported for the consensus refinement, thus allowing classification on high resolution features. Several T values ranging from 20 to 70 were empirically tested and a T value of 40 was found to give the best results. A single class, containing 27,310 particles, was identified that possessed well resolved density for the C-terminal domain of SGBP^lev^. A particle star file containing information for particles contributing to this class was created manually via command line arguments. From this, two new star files were generated that contained random half sets of the selected data. Using relion_reconstruct, these star files were used to generate two independent half maps that corresponded to the unmasked structure. Post-processing using these generated half maps yielded a sharpened reconstruction of 3.0 Å, as estimated by gold standard Fourier Shell correlations using the 0.143 criterion. The density for the C-terminal domain of the SGBP^lev^ was improved, and density corresponding to levan chain that links SGBP^lev^ to the GH^lev^ was also visible.

To obtain the highest quality density for FOS molecules occupying the SusCD^lev^ binding cavity, all particles were considered regardless of lipoprotein complement or conformation. A particle subtraction and focused refinement strategy targeting the SusCD^lev^ core of the complex was used as described for the active levan utilisome. Post-processing of the model arising from this final C2 symmetrised refinement resulted in a sharpened reconstruction at 2.7 Å.

### Dextran utilisome cryo-EM sample preparation, data collection and image processing

A sample of the dextran utilisome complex (Bt3087-Bt3090) solubilised in a DDM-containing buffer (10 mM HEPES, 100 mM NaCl, pH 7.5, 0.03 % DDM) was prepared at 0.05 mg/ml. Lacy carbon 300-mesh copper grids coated with a <3 nm continuous carbon film (Agar Scientific) were glow-discharged in air (10 mA, 30 s). A sample volume of 3.5 mL was applied to the grid. Blotting and plunge-freezing were performed 10 seconds after loading the sample onto the grid using an FEI Vitrobot Mark IV (Thermo Fisher Scientific) with chamber conditions set at a temperature of 4 °C and 100% relative humidity. The grid was blotted for 6 s with a

blot force of 0. Micrograph movies were collected on a Titan Krios Microscope (Thermo Fisher Scientific) operating at 300 kV with a Falcon IV direct electron detector operating in counting mode. Data acquisition parameters can be found in Supplementary Table 5.

A dataset comprising 6,331 micrographs was collected and image processing was carried out in RELION 3.1 ^55^. Drift correction and dose-weighting were performed using RELION’s own implementation of MOTIONCOR2. CTF estimation of motion corrected micrographs was done using CTFFIND4 ^60^. Particle picking was done using the crYOLO general model which identified 820,184 particles in the micrographs. Junk particles and contaminants were removed through several rounds of 2D classification, after which 477,707 particles remained. An initial model was generated *de novo* from the data. Extensive 3D classification was used to address the considerable compositional heterogeneity that was present in the data (see ED Fig. 6). Each unique composition was refined and sharpened independently. A consensus refinement was carried out, with iterative rounds of CTF-refinement and Bayesian polishing until no further improvement in resolution was seen. A final, sharpened consensus reconstruction was obtained at 3.1 Å.

### Model building into electron microscopy maps

Buccaneer (part of CCP-EM v1.5.0)^61,62^ was used to build the initial protein models into the post-processed consensus inactive levanase map, resulting in almost complete protein models. An AlphaFold2 ^23^ prediction of the dextran SusC was generated and used as an initial model. Manual modelling and real space refinement of protein and FOS chains were performed iteratively using COOT^52^ and Phenix^54^, respectively. The completed protein and FOS models were placed into other maps and real space refined. The acyl-cysteine was designated as the first residue of each lipoprotein. Model refinement statistics are presented in Supplementary Tables 2-5.

### Density analysis and figure making

Investigation and comparison of EM density maps was performed using Chimera^59^ and COOT^52^. Figures of maps and models were generated using Chimera and ChimeraX^63^. To aid interpretability of EM density in generated figures, some maps were filtered using LAFTER^64^. Maps processed in this way are clearly indicated in the corresponding figure legend. The masks supplied in filtering were the same masks used for post-processing within RELION.

## Extended Data

## Supplementary Material

Source Data

Supplementary Material

Supplementary Materials

## Data Availability

The data supporting the findings of this study are available from the corresponding authors upon reasonable request. Cryo-EM reconstructions and corresponding coordinates have been deposited in the Electron Microscopy Data Bank and the Protein Data Bank respectively: Substrate free levan utilisome (EMD-15288, PDB ID 8A9Y), levan utilisome with FOS DP 8-12 (EMD-15289, PDB ID 8AA0), SusC_2_D_2_ core from the levan utilisome with FOS DP 8-12 (EMD-15290, PDB ID 8AA1), inactive levan utilisome with FOS DP 15-25 (EMD-15291, PDB ID 8AA2), SusC_2_D_2_ core from inactive levan utilisome with FOS DP 15-25 (EMD-1592, PDB ID 8AA3), dextran utilisome consensus refinement (EMD-15293, PDB ID 8AA4). Raw cryo-EM movies will be deposited in the EMPIAR database. Coordinates and structure factors from X-ray crystallography experiments for GH^lev^ have been deposited in the Protein Data Bank under the accession codes 7ZNR and 7ZNS. The mass spectrometry proteomics data have been deposited to the ProteomeXchange Consortium via the PRIDE partner repository with the dataset identifier PXD034863. The reviewer account details for the proteomics data are as follows: **Username:** reviewer_pxd034863@ebi.ac.uk; **Password:** 0YCD9u3J.
