# Supplementary material for: Outer membrane utilisomes mediate oligosaccharide uptake in gut Bacteroidetes
Source: Nature. Author manuscript; Available in PMC 2025 Dec 9. (PMC7618045; doi:10.1038/s41586-023-06146-w)
Supplement: Supplementary Material [file EMS208029-supplement-Supplementary_Material.zip › 41586_2023_6146_MOESM2_ESM.pdf]

nature portfolio

Corresponding author(s): Neil A Ranson & Bert van den Berg

Last updated by author(s): 31st March 2023

Reporting Summary

Nature Portfolio wishes to improve the reproducibility of the work that we publish. This form provides structure for consistency and transparency in reporting. For further information on Nature Portfolio policies, see our Editorial Policies and the Editorial Policy Checklist.

Statistics

For all statistical analyses, confirm that the following items are present in the figure legend, table legend, main text, or Methods section.

n/a	Confirmed	
☐	☒	The exact sample size (*n*) for each experimental group/condition, given as a discrete number and unit of measurement
☐	☒	A statement on whether measurements were taken from distinct samples or whether the same sample was measured repeatedly
☐	☒	The statistical test(s) used AND whether they are one- or two-sided<br>*Only common tests should be described solely by name; describe more complex techniques in the Methods section.*
☒	☐	A description of all covariates tested
☐	☒	A description of any assumptions or corrections, such as tests of normality and adjustment for multiple comparisons
☒	☐	A full description of the statistical parameters including central tendency (e.g. means) or other basic estimates (e.g. regression coefficient) AND variation (e.g. standard deviation) or associated estimates of uncertainty (e.g. confidence intervals)
☐	☒	For null hypothesis testing, the test statistic (e.g. F, t, r) with confidence intervals, effect sizes, degrees of freedom and P value noted<br>*Give P values as exact values whenever suitable.*
☒	☐	For Bayesian analysis, information on the choice of priors and Markov chain Monte Carlo settings
☒	☐	For hierarchical and complex designs, identification of the appropriate level for tests and full reporting of outcomes
☒	☐	Estimates of effect sizes (e.g. Cohen's *d*, Pearson's *r*), indicating how they were calculated

Our web collection on statistics for biologists contains articles on many of the points above.

Software and code

Policy information about availability of computer code

Data collection	X-ray crystallography:<br>XIA2-DIALS -<br>AIMLESS<br><br>CryoEM:<br>EPU 1.20.3.10
Data analysis	Isothermal Titration Calorimetry:<br>Microcal PEAQ-ITC analysis software v1.40<br><br>Mass Spec & Proteomics:<br>MaxQuant 1.6.14.0<br>R-Limma 3.48.0<br><br>X-ray crystallography:<br>XDS: 20210205<br>Pointless 1.12.12<br>Crank2 2.0.281<br>CCP4cloud - 1.7..007<br>CCP4 build - 1.0.3

Coot v0.9.8.1
Refmac: 5.8.0267

CryoEM:
Relion 3.1
Cryolo 1.6.1
CCPEM 1.5.1-buccaneer
CCPEM 1.51-LAFTER
Coot v0.9.8.1
Phenix v1.20rc3-4406
UCSF Chimera 1.13.1
UCSF ChimeraX 0.91

Structure Prediction:
Alphafold2

For manuscripts utilizing custom algorithms or software that are central to the research but not yet described in published literature, software must be made available to editors and reviewers. We strongly encourage code deposition in a community repository (e.g. GitHub). See the Nature Portfolio guidelines for submitting code & software for further information.

Data

Policy information about availability of data

All manuscripts must include a data availability statement. This statement should provide the following information, where applicable:

- Accession codes, unique identifiers, or web links for publicly available datasets
- A description of any restrictions on data availability
- For clinical datasets or third party data, please ensure that the statement adheres to our policy

The data supporting the findings of this study are available from the corresponding authors upon reasonable request. Cryo-EM reconstructions and corresponding coordinates have been deposited in the Electron Microscopy Data Bank and the Protein Data Bank respectively: Substrate free levan utilisome (EMD-15288, PDB ID 8A9Y), levan utilisome with FOS DP 8-12 (EMD-15289, PDB ID 8AA0), SusC2D2 core from the levan utilisome with FOS DP 8-12 (EMD-15290, PDB ID 8AA1), inactive levan utilisome with FOS DP 15-25 (EMD-15291, PDB ID 8AA2), SusC2D2 core from inactive levan utilisome with FOS DP 15-25 (EMD-1592, PDB ID 8AA3), dextran utilisome consensus refinement (EMD-15293, PDB ID 8AA4). Raw cryo-EM movies will be deposited in the EMPIAR database. Coordinates and structure factors from X-ray crystallography experiments for GHlev have been deposited in the Protein Data Bank under the accession codes 7ZNR and 7ZNS. The mass spectrometry proteomics data have been deposited to the ProteomeXchange Consortium via the PRIDE partner repository with the dataset identifier PXD034863. The reviewer account details for the proteomics data are as follows: Username: reviewer_pxd034863@ebi.ac.uk; Password: 0YCD9u3J.

Human research participants

Policy information about studies involving human research participants and Sex and Gender in Research.

Reporting on sex and gender	N/A
Population characteristics	N/A
Recruitment	N/A
Ethics oversight	N/A

Note that full information on the approval of the study protocol must also be provided in the manuscript.

Field-specific reporting

Please select the one below that is the best fit for your research. If you are not sure, read the appropriate sections before making your selection.

☒ Life sciences ☐ Behavioural & social sciences ☐ Ecological, evolutionary & environmental sciences

For a reference copy of the document with all sections, see nature.com/documents/nr-reporting-summary-flat.pdf

Life sciences study design

All studies must disclose on these points even when the disclosure is negative.

Sample size	No statistical methods were used to determine sample size.
Data exclusions	No data were excluded from the analyses

Replication	ITC experiments were repeated at least twice and all attempts at replication were successful. For proteomics experiments, three biological replicates of each condition were used. Structural experiments were not repeated.
Randomization	Designation of experimental groups was not performed in this study. The biochemical and biophysical analyses performed were ensemble techniques measuring the properties/behaviours of random populations of particles/proteins/peptides.
Blinding	Group allocation was not involved in this study and therefore comments on blinding are not applicable.

Reporting for specific materials, systems and methods

We require information from authors about some types of materials, experimental systems and methods used in many studies. Here, indicate whether each material, system or method listed is relevant to your study. If you are not sure if a list item applies to your research, read the appropriate section before selecting a response.

Materials & experimental systems

n/a	Involved in the study	
☒	☐	Antibodies
☒	☐	Eukaryotic cell lines
☒	☐	Palaeontology and archaeology
☒	☐	Animals and other organisms
☒	☐	Clinical data
☒	☐	Dual use research of concern

Methods

n/a	Involved in the study	
☒	☐	ChIP-seq
☒	☐	Flow cytometry
☒	☐	MRI-based neuroimaging
