# Supplementary material for: Outer membrane utilisomes mediate oligosaccharide uptake in gut Bacteroidetes
Source: Nature. Author manuscript; Available in PMC 2025 Dec 9. (PMC7618045; doi:10.1038/s41586-023-06146-w)
Supplement: Supplementary Materials [file EMS208029-supplement-Supplementary_Materials.pdf]

Supplementary Discussion

TonB-dependent transport and signalling substrate binding across the OM

TonB-dependent transporters (TBDTs), including SusC-type TBDTs, require energy for their transport activity. Because there are no ion gradients across the OM nor ATP in the periplasm, TBDTs couple to an inner membrane (IM) complex that harnesses the energy stored in the proton gradient across the IM. In the latest model for transport via TBDTs[1] (Supplementary Figure 1), protons are conducted by the ExbBD complex into the cytoplasmic space, which induces rotation or movement of the IM TonB protein that is most likely bound to the outside of the ExbBD complex[1]. The C-terminal domain of TonB binds to the TonB box on the periplasmic face of the TBDT in the presence of substrate. In this way, TonB transduces mechanical energy from the inner membrane to the TBDT in a poorly-understood process that results in partial unfolding of the TBDT barrel plug and, consequently, the formation of a channel via which the substrate can diffuse into the periplasm (Supplementary Figure 1)[2]. Following transport, the TonB C-terminal domain (CTD) interaction with the TonB box is broken via an unknown mechanism, after which the plug re-folds and closes the substrate channel, resetting the transporter for another cycle.

A key feature of the TBDT transport mechanism is prevention of unproductive transport cycles, *i.e*. engagement of TonB with TBDTs that do not have any substrate bound. Information about substrate binding on the extracellular side of the TBDT must be relayed across the OM to the periplasm. This is achieved via substrate binding-induced conformational changes that are propagated through the barrel and the plug of the TBDT. This ultimately leads to increased exposure of the TonB box in the periplasmic space, which is a pre-requisite for interaction with the TonB CTD, disruption of the plug domain and consequent channel formation. In the *E. coli* vitamin B12 transporter BtuB for example, a salt bridge, or 'ionic lock', is present between the barrel wall and a plug residue downstream from the TonB box in the absence of substrate. Binding of vitamin B12 by extracellular loops and the apex of the plug causes allosteric conformational changes, breaking the ionic lock and increasing accessibility of the TonB box[3,4]. However, while statistical coupling analysis (SCA) and structure-based analyses have provided useful insights[5,6], identifying the exact residues involved in these conformational changes upon substrate binding has been challenging, as TBDT structures with and without substrate are usually very similar beyond the TonB box[7].

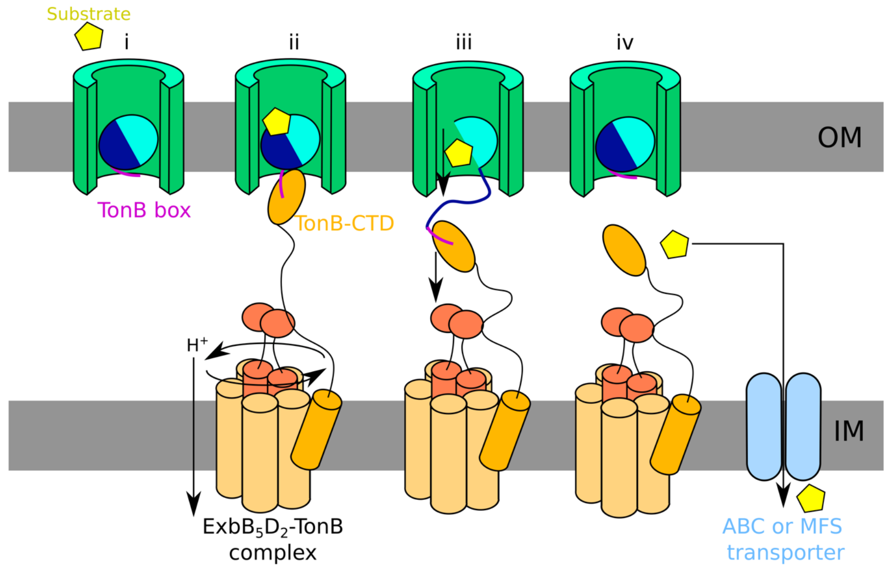

Supplementary Figure 1. The TonB-dependent transport cycle. (i) The β-barrel of the TonB-dependent transporter (green) is occluded by the plug domain (blue/cyan) when no substrate is bound. The TonB box is poorly accessible from the periplasm. (ii) Substrate binding induces conformational changes in the TonB-dependent transporter that lead to increased periplasmic accessibility of the TonB box. The TonB CTD binds the exposed TonB box. The transmembrane segment of TonB is associated with the proton-conducting IM ExbBD complex. (iii) TonB transduces the energy stored in the proton gradient to exert a force on the mechanically-labile subdomain of the plug (dark blue), which is pulled out of the β-barrel lumen. The substrate can diffuse into the periplasm via the open transport channel. (iv) TonB disengages the TonB box, the mechanically labile subdomain of the plug re-inserts into the β-barrel lumen, and the substrate is further processed in the periplasm (in the case of *e.g.* oligosaccharides; not shown) and imported into the cytoplasm via ATP binding cassette (ABC) or major facilitator superfamily (MFS) transporters. A "classical" TBDT such as *E. coli* BtuB is shown for simplicity. As such, lipoprotein components of the utilisome are not shown. Likewise, the N-terminal extension (NTE) domain, present N-terminal to the TonB box in many SusC transporters, is not shown.

While our cryo-EM structures of the apo- and FOS-bound levan utilisomes shed some light on the allosteric signalling pathway within SusC^{lev}, experimentally proving that the residues that undergo conformational changes upon substrate binding indeed form an allosteric network remains profoundly challenging. Part of this stems from the fact that, so far, it has not been possible to reconstitute TonB-dependent transport *in vitro*, leaving growth assays as the only way to test the effect of mutations within the allosteric network. Moreover, such mutations would generate negative results (*i.e.*, no growth on levan). In principle, progress might be made via methods that allow interrogation of protein dynamics on relevant timescales, *e.g.* in vivo electron paramagnetic resonance or hydrogen-deuterium exchange mass spectrometry

which have only recently been applied to studying BtuB[3,4]. Adapting such methods to the anaerobic *B. theta* will be a challenge.

The presence of one, or sometimes two additional N-terminal domains in SusC-type TBDTs represents an additional layer of complexity compared to classical TBDTs such as *E. coli* FhuA or BtuB. STN domains, absent in both $SusC^{lev}$ and $SusC^{dex}$, are involved in cell surface signalling by interacting with IM-embedded anti-sigma regulators[8,9]. An N-terminal extension (NTE) domain is present N-terminal and adjacent to the TonB box in most *B. theta* TBDTs, including $SusC^{lev}$ and $SusC^{dex}$. It is approximately 7 kDa in size and has an Ig-like fold[10]. The function of the NTE is not known, although its location suggests it could plausibly interact with the TonB CTD in the periplasmic space. Intriguingly, deletion of the NTE in $SusC^{lev}$ and *P. gingivalis* RagA generates a growth defect that is much more severe than a deletion of the TonB box, suggesting a role for the NTE that goes beyond the transport process itself[10].

Glycan specificity of the $SGBP^{lev}$

Investigation of the levan binding site of $SGBP^{lev}$ (ED Fig. 10) reveals three tryptophan residues (W297, W311 and W359) that potentially form stacking interactions with the β-fructofuranose units of the β2,6-linked FOS chain, consistent with previous SGBP-ligand complex structures[11,12]. Aromatic stacking makes a crucial contribution to binding affinity as demonstrated by ITC with alanine substitutions of the $SGBP^{lev}$ (ED Fig. 9a). Importantly, the orientation of the aromatic side chains affects the shape of the binding site. This results in specificity by enabling stacking interactions between the aromatic side chains and the β-fructofuranose faces only in a β2,6-linked FOS chain. Indeed, our ITC data clearly show that $SGBP^{lev}$ has a relatively high affinity for levan but does not bind another fructan, β2,1-linked inulin (ED Fig. 9d).

Given that the local resolution of our $SGBP^{lev}$ maps was insufficient to assign hydrogen bonds with confidence, we performed a BLAST search with the $SGBP^{lev}$ C-terminal domain amino acid sequence and made an alignment with sequences that were 90%, 47%, 33% and 23% identical to $SGBP^{lev}$ (Supplementary Figure 2). We confirmed that all chosen sequences are likely to be genuine levan SGBPs by looking at their genomic context: all of the sequences are part of a PUL that also has a gene predicted to encode a GH32 family enzyme (endo-levanase). Through analysis of AlphaFold2-predicted models[13] of these $SGBP^{lev}$ homologues, we infer that a number of residues are also involved in specific hydrogen-bonding interactions with FOS (N295, T350, Q352, N384) (ED Fig. 10). These binding site residues are mostly conserved, even in proteins with only ~33% sequence identity to that of $SGBP^{lev}$ (ED Fig 10, Supplementary Figure 2). It is likely that these hydrogen-bonding residues, as well as the

stacking tryptophan residues, are configured within the binding site in such a way that allows them to bind only glycans with the correct chemical composition as well as the correct geometry or secondary structure imposed by a specific glycosidic linkage. Other highly conserved residues that do not bind levan are likely involved in maintaining the fold of the domain.

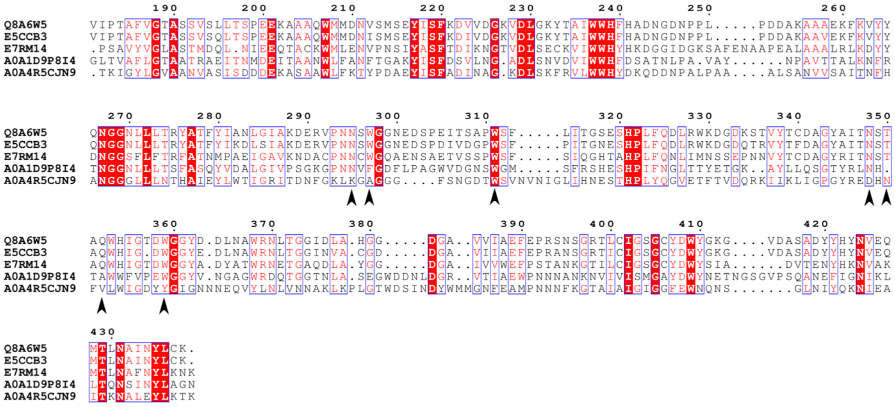

Supplementary Figure 2. SGBP^{lev} C-terminal domain homologue amino acid sequence alignment. Only the C-terminal levan binding domain sequences of the SGBP^{lev} were aligned. UniProt accession numbers correspond to: *B. theta* VPI-5482 SGBP^{lev} (Q8A6W5); *Bacteroides* sp. D2 (E5CCB3); *Prevotella oralis* ATCC 33269 (E7RM14); *Flavobacterium commune* (A0A1D9P8I4); *F. cellulosilyticum* (A0A4R5CJN9). Residue numbering is for *B. theta* SGBP^{lev}. Arrowheads indicate positions in the *B. theta* SGBP^{lev} sequence that bind to FOS in the cryo-EM structure. The alignment was made with Clustal Omega[14], and visualised in ESPript 3.0[15].

References

1. Ratliff, A. C., Buchanan, S. K. & Celia, H. The Ton Motor. *Front. Microbiol.* **13**, 1240 (2022).
2. Hickman, S. J., Cooper, R. E. M., Bellucci, L., Paci, E. & Brockwell, D. J. Gating of TonB-dependent transporters by substrate-specific forced remodelling. *Nat. Commun. 2017 81* **8**, 1–12 (2017).
3. Nilaweera, T. D., Nyenhuis, D. A. & Cafiso, D. S. Structural intermediates observed only in intact Escherichia coli indicate a mechanism for TonB-dependent transport. *Elife* **10**, (2021).
4. Zmyslowski, A. M., Baxa, M. C., Gagnon, I. A. & Sosnick, T. R. HDX-MS performed on BtuB in E. coli outer membranes delineates the luminal domain's allostery and unfolding upon B12 and TonB binding. *Proc. Natl. Acad. Sci. U. S. A.* **119**, (2022).
5. Ferguson, A. D. *et al.* Signal transduction pathway of TonB-dependent transporters. *Proc. Natl. Acad. Sci. U. S. A.* **104**, 513–518 (2007).
6. Chimento, D. P., Kadner, R. J. & Wiener, M. C. Comparative structural analysis of TonB-dependent outer membrane transporters: Implications for the transport cycle. *Proteins Struct. Funct. Genet.* **59**, 240–251 (2005).
7. Noinaj, N., Guillier, M., Barnard, T. J. & Buchanan, S. K. TonB-Dependent Transporters: Regulation, Structure, and Function. *Annu. Rev. Microbiol.* **64**, 43–60 (2010).
8. Malki, I. *et al.* Interaction of a Partially Disordered Antisigma Factor with Its Partner, the Signaling Domain of the TonB-Dependent Transporter HasR. *PLoS One* **9**, e89502 (2014).
9. Jensen, J. L., Jernberg, B. D., Sinha, S. & Colbert, C. L. Structural basis of cell surface signaling by a conserved sigma regulator in Gram-negative bacteria. *J. Biol. Chem.* jbc.RA119.010697 (2020). doi:10.1074/jbc.RA119.010697
10. Gray, D. A. *et al.* Insights into SusCD-mediated glycan import by a prominent gut symbiont. *Nat. Commun.* **12**, 1–14 (2021).
11. Tamura, K. *et al.* Surface glycan-binding proteins are essential for cereal beta-glucan utilization by the human gut symbiont Bacteroides ovatus. *Cell. Mol. Life Sci.* **76**, 4319–4340 (2019).
12. Tamura, K., Dejean, G., Van Petegem, F. & Brumer, H. Distinct protein architectures mediate species-specific beta-glucan binding and metabolism in the human gut microbiota. *J. Biol. Chem.* **296**, (2021).
13. Jumper, J. *et al.* Highly accurate protein structure prediction with AlphaFold. *Nat. 2021 5967873* **596**, 583–589 (2021).
14. Sievers, F. *et al.* Fast, scalable generation of high-quality protein multiple sequence alignments using Clustal Omega. *Mol. Syst. Biol.* **7**, 539 (2011).
15. Robert, X. & Gouet, P. Deciphering key features in protein structures with the new ENDscript server. *Nucleic Acids Res.* **42**, (2014).

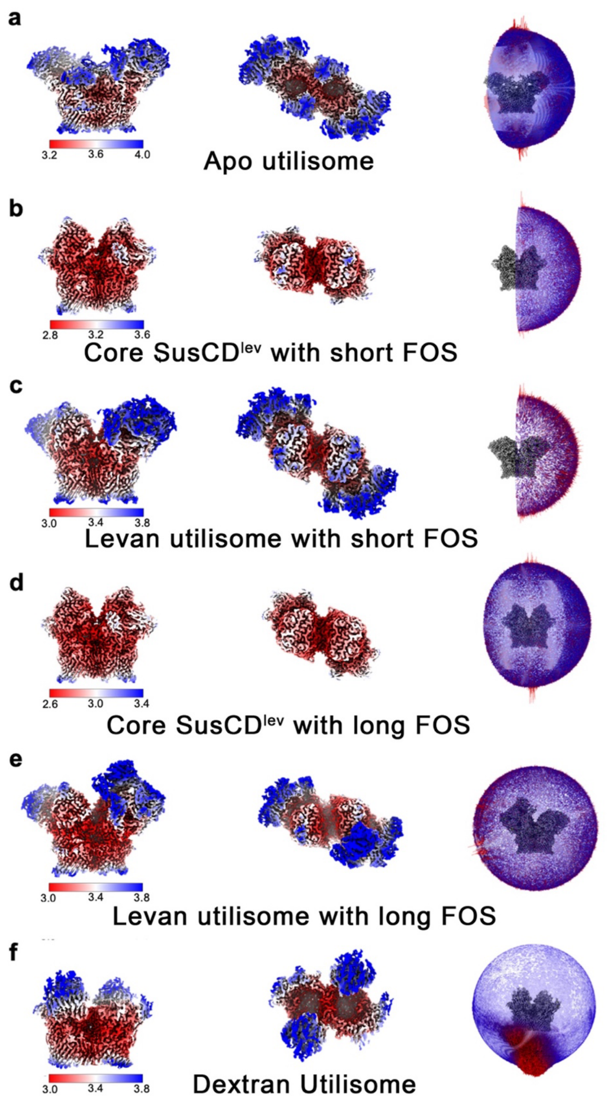

Supplementary Figure 3. **Reconstructions of levan and dextran systems filtered and coloured by local resolution.** Side view (left column), and top view (centre column) of the various utilisome maps (and sub-complexes) presented in this work. All maps are coloured according to the colour key accompanying each. Right hand column shows angular distribution plots for each reconstruction, and highlights the strongly preferred orientation of the Dextran Utilisome data.

Supplementary Table 1. Crystallography data statistics and refinement details.

Data statistics*			
	BT1760 SeMet	BT1760 D42N	BT1760 D42N
Beamline	I03	I02	I04
Date	05/08/08	27/09/09	31/05/10
Wavelength (Å)	0.97630	0.97930	0.97930
Resolution (Å)	61.92-2.80 (2.85-2.80)	67.87-2.65 (2.74-2.65)	73.71-2.30 (2.35-2.30)
Space group	$P4_12_12$	$I4_122$	$I4_122$
Unit-cell parameters			
a (Å)	175.14	174.72	175.50
b (Å)	175.14	174.72	175.50
c (Å)	221.50	215.59	214.53
α (°)	90.0	90.0	90.0
β (°)	90.0	90.0	90.0
γ (°)	90.0	90.0	90.0
Unit-cell volume ($Å^3$)	6794525	6581332	6607577
Solvent content (%)	49	47	47
No. of measured reflections	4649806 (166168)	788798 (66908)	512272 (18602)
No. of independent reflections	83001 (3468)	48504 (4407)	73764 (4314)
Completeness (%)	97.7 (76.0)	99.9 (99.7)	99.6 (95.4)
Redundancy	56.0 (47.9)	16.3 (15.2)	6.9 (4.3)
$CC_{1/2}$ (%)	99.8 (87.9)	99.7 (70.8)	99.5 (54.9)
$I/\sigma(I)$	22.7 (4.3)	12.1 (1.2)	9.4 (1.1)
Anomalous completeness (%)	97.8 (76.3)		
Anomalous redundancy	29.3 (24.6)		
Refinement Statistics			
Rwork (%)		19.13	20.89
Rfree[#] (%)		25.55	26.75
No. of non-H atoms			
No. of protein atoms		7800	7800
No. of solvent atoms		5	201
No. of ligand atoms		284	230
R.m.s. deviation from ideal			
Bond angle (°)		2.14	1.89
Bond length (Å)		0.0099	0.0094
Average B factor ($Å^2$)			
Protein		59	62
Solvent		38	54
Ligand		81	77
Ramachandran plot[+], residues in most favoured regions (%)		91.2	92.9
PDB file code		7ZNR	7ZNS

*(Values in parentheses are for the highest resolution shell).

[#]5% of the randomly selected reflections excluded from refinement.

[+]Calculated using MOLPROBITY.

Supplementary Table 2. Data acquisition parameters for the two levan four-component complex datasets obtained in the absence of substrate. Datasets were combined and refinement statistics for the model of the levan utilisome complex are shown. Note that SusD subunits were not included in this model.

	Dataset 1	Dataset 2
**Data collection and processing**		
Magnification	75,000 x	75,000 x
Voltage (kV)	300	300
Electron exposure (e^-/Å^2)	35.4	37
Defocus range (μm)	-1.5 to -3.3	-1.5 to -3.3
Pixel size (Å)	1.065	1.065
Initial particle images pre-3D classification (no.)	89,305	280,696
Final utilisome particles post-classification (no.)	45,594	146,512
Total utilisome particles after datasets combined (no.)	192,106	
Symmetry imposed	C2	
Map resolution (Å) (FSC 0.143)	3.5	
Map sharpening B-factor (Å^2)	-149	
**Refinement**		
Model composition		
Non-hydrogen atoms	23,688	
Protein residues	2988	
R.m.s. deviations		
Bond lengths (Å)	0.002	
Bond angles (°)	0.479	
Validation		
Molprobity score	1.75	
Clashscore	8.48	
Poor rotamers (%)	0	
Ramachandran plot		
Favoured (%)	95.76	
Allowed (%)	4.17	
Disallowed (%)	0.07	
**Deposition ID**		
PDB	8A9Y	
EMDB	EMD-15288	

Supplementary Table 3. Data acquisition parameters for the levan four-component complex in the presence of FOS DP8-12, and refinement statistics for models of the levan utilisome complex and $SusC_2D_2$ core.

**Data collection and processing**		
Magnification		
Voltage (kV)	300	
Electron exposure ($e^-/Å^2$)	38.5	
Defocus range (µm)	-1.5 to -3.0	
Pixel size (Å)	1.065	
Initial particle images pre-3D classification (no.)	63,789	
Particle stack	**Utilisome**	**$SusC_2D_2$ core**
Final particle images post-classification (no.)	15,012	54,736
Symmetry imposed	C2	C2
Map resolution (Å) (FSC 0.143)	3.2	2.9
Map sharpening B-factor ($Å^2$)	-90.63	-96.42
**Refinement**		
Model composition		
Non-hydrogen atoms	33,058	23,634
Protein residues	4134	2950
R.m.s. deviations		
Bond lengths (Å)	0.005	0.002
Bond angles (°)	0.555	0.459
Validation		
Molprobity score	1.75	1.38
Clashscore	7.80	6.98
Poor rotamers (%)	0	0
Ramachandran plot		
Favoured (%)	95.34	98.37
Allowed (%)	4.47	1.56
Disallowed (%)	0.19	0.07
**Deposition ID**		
PDB	8AA0	8AA1
EMDB	EMD-15289	EMD-15290

Supplementary Table 4. Data acquisition parameters for the levan four-component complex with inactivated levanase in the presence of FOS DP15-25 and refinement statistics for models of the utilisome with a tethered conformation Bt1761, and of the $SusC_2D_2$ core.

**Data collection and processing**		
Magnification	75,000 x	
Voltage (kV)	300	
Electron exposure ($e^-/Å^2$)	37.8	
Defocus range (μm)	-1.5 to -3.0	
Pixel size (Å)	1.065	
Initial particle images pre-3D classification (no.)	146,056	
Particle stack	**From focused 3D incl. resolved Bt1761**	**Containing $SusC_2D_2$ core**
Final particle images post-classification (no.)	27,310	120,957
Symmetry imposed	C1	C2
Map resolution (Å) (FSC 0.143)	3.1	2.7
Map sharpening B-factor ($Å^2$)	-76.41	-91.93
**Refinement**		
Model composition		
Non-hydrogen atoms	35,942	23,634
Protein residues	4472	2950
R.m.s. deviations		
Bond lengths	0.003	0.001
Bond angles (°)	0.486	0.397
Validation		
Molprobity score	1.62	1.38
Clashscore	7.25	6.90
Poor rotamers (%)	0	0
Ramachandran plot		
Favoured (%)	96.57	98.37
Allowed (%)	3.37	1.63
Disallowed (%)	0.07	0
**Deposition ID**		
PDB	8AA2	8AA3
EMDB	EMD-15291	EMD-1592

Supplementary Table 5. Data acquisition parameters for the dextran four-component complex and refinement statistics for a model of the dimeric dextran transporting SusC (Bt3090). Map anisotropy owing to suboptimal angular distribution of particles meant density was of insufficient quality to build the SusD components.

**Data collection and processing**	
Magnification	96,000 x
Voltage (kV)	300
Electron exposure (e^-/Å^2)	38.78
Defocus range (μm)	-1.2 to -3.0
Pixel size (Å)	0.86
Initial particle images pre-3D classification (no.)	477,707
Final particle images post-classification (no.)	305,372
Symmetry imposed	C1
Map resolution (FSC 0.143)	3.1
Map sharpening B-factor (Å^2)	-79.2
**Refinement**	
Model composition	
Non-hydrogen atoms	13,616
Protein residues	1742
R.m.s. deviations	
Bond lengths (Å)	0.007
Bond angles (°)	0.672
Validation	
Molprobity score	2.24
Clashscore	4.8
Poor rotamers (%)	4.69
Ramachandran plot	
Favoured (%)	92.08
Allowed (%)	7.92
Disallowed (%)	0
**Deposition ID**	
PDB	8AA4
EMDB	EMD-15293
